# Integrated analysis of an in vivo model of intra-nasal exposure to instilled air pollutants reveals cell-type specific responses in the placenta

**DOI:** 10.1038/s41598-022-12340-z

**Published:** 2022-05-19

**Authors:** Anela Tosevska, Shubhamoy Ghosh, Amit Ganguly, Monica Cappelletti, Suhas G. Kallapur, Matteo Pellegrini, Sherin U. Devaskar

**Affiliations:** 1 Department of Molecular, Cell and Developmental Biology, University of California Los Angeles, Los Angeles, CA USA; 2 Division of Neonatology & Developmental Biology, Department of Pediatrics, and the UCLA Children’s Discovery & Innovation Institute, David Geffen School of Medicine at University of California Los Angeles, 10883, Le Conte Avenue, MDCC-22-412, Los Angeles, CA 90095-1752 USA; 3 Present Address: Division of Rheumatology, Internal Medicine III, Medical University of Vienna, Vienna, Austria

**Keywords:** Molecular biology, Physiology, Molecular medicine

## Abstract

The placenta is a heterogeneous organ whose development involves complex interactions of trophoblasts with decidual, vascular, and immune cells at the fetal–maternal interface. It maintains a critical balance between maternal and fetal homeostasis. Placental dysfunction can lead to adverse pregnancy outcomes including intra-uterine growth restriction, pre-eclampsia, or pre-term birth. Exposure to environmental pollutants contributes to the development of placental abnormalities, with poorly understood molecular underpinning. Here we used a mouse (C57BL/6) model of environmental pollutant exposure by administration of a particulate matter (SRM1649b at 300 μg/day/mouse) suspension intra-nasally beginning 2 months before conception and during gestation, in comparison to saline-exposed controls. Placental transcriptomes, at day 19 of gestation, were determined using bulk RNA-seq from whole placentas of exposed (n = 4) and control (n = 4) animals and scRNAseq of three distinct placental layers, followed by flow cytometry analysis of the placental immune cell landscape. Our results indicate a reduction in vascular placental cells, especially cells responsible for structural integrity, and increase in trophoblast proliferation in animals exposed to particulate matter. Pollution-induced inflammation was also evident, especially in the decidual layer. These data indicate that environmental exposure to air pollutants triggers changes in the placental cellular composition, mediating adverse pregnancy outcomes.

## Introduction

The impact of the intra-uterine environment upon the conceptus can be far reaching, given that changes incurred during this vulnerable and critical developmental period have long standing implications with changes of permanency. Much of this adaptive imprint is delivered to developing tissues by changes in gene expression and cellular content. The placenta, an organ that co-habitats with the conceptus, acts as a functional interface delivering oxygen and nutrients to the developing fetus^1,2^. In addition, the placenta produces a plethora of hormones necessary for the physiological adaptation necessary during pregnancy^1,3^. The placenta also serves as an effective barrier protecting the fetus from toxins and pathogens^1,4^. Since the placenta co-exists with the fetus, it also encounters various intra-uterine exposures, and experiences certain detrimental effects, which in turn could negatively impact maintenance of a pregnancy or fetal health^2^. Such imposition of placental changes ultimately adversely affects the well-being of mother and conceptus^2^. Environmental exposures from air pollution, especially particulate matter originating in urban areas can have far-reaching effects on maternal well-being and fetal development^5–7^, and have been implicated in adverse pregnancy outcomes, such as preterm labor^8^, gestational diabetes mellitus^9^ or pre-eclampsia^10^ presenting with fetal growth restriction and low birth weights^8,11,12^. In order to understand the effects of environmental exposures on mother and conceptus, it is important to study the placenta. In particular one needs to understand the complex cellular and signaling patterns underlying placental development.

Tissues, including the placenta, are comprised of diverse cell types with distinguishable developmental or functional origin that form a complex niche^1^. A comprehensive assessment of cellular heterogeneity is traditionally performed by immunophenotyping which can be biased and relies on a small set of pre-selected markers, limiting the cell types that can be inspected^13^. Alternatively, Bulk RNA sequencing (RNA-seq) offers an unbiased approach to tissue profiling with greater resolution, accounting for the dynamic nature of the transcriptome^14^. RNA-seq can also help to identify novel transcripts, alternately spliced genes, and allele-specific expression. However, RNA-seq typically represents an average of gene expression across millions of cells which may obscure cellular heterogeneity, especially in organs or tissues with multiple cell types^15^. To overcome this barrier, one could isolate single cells and capture their transcripts by employing single cell RNA-seq, a technique that can assess the cell population structure in depth and the nuances of various cell signaling pathways with unprecedented resolution^16^. Single cell transcriptomics performed by 10X genomics is an established technology that is helpful in deciphering cell-specific gene expression in complex organs and tissues subjected to various environmental stressors^17^. The introduction of this technology has proven to be highly useful in unraveling the major and minor cell types present in a sample, in addition to identifying differentially expressed genes. This has resulted in the discovery of various novel cell types and their abundance under normal and pathological circumstances^18,19^.

Previous studies have examined the human placenta during the first trimester and provided information regarding the cellular content and transcriptomics under normal circumstances^20,21^ and in the presence of pre-eclampsia^20^. Similarly, murine placental scRNA-sequencing has been performed under normal circumstances^22–24^. However, given distinct differences between human and murine placentas^25^ and the fact that manipulations during human pregnancy are not possible, the development of genetic and pathological murine pregnancy models has been applied to various conditions. Examples include intrauterine growth restriction^26^ due to maternal calorie restriction^27^, maternal diabetes^28^ and hypertension^27^, among others. More recently, the effect on pregnancy of exposures to air pollutants has gained increasing visibility resulting in the development of multiple mouse models^29,30^. Using one such nasal instillation gestational model, we sought to assess the impact of air pollutants on the late gestation placental transcriptomics and cellular composition. We hypothesized that employing single-cell transcriptomics and deconvolution of placental bulk transcriptomics will provide the basis for identification of cellular composition, setting the stage for application to various murine models beyond the one we have tested here. To test this hypothesis, we studied the late gestational placentas divided into three layers, namely the decidual, junctional and labyrinthine layers and identified the cellular and gene expression signatures.

## Results

### Differential abundance of cell types observed by scRNAseq in air pollution exposed placentas versus controls

Six animals (3 exposed to air pollution and 3 controls) were used for scRNAseq analysis. Placentas were collected from each pregnancy, separated into 3 placental layers and tissue from the same placental layers were pooled for each group, yielding a total of six samples (two treatments and three layers). A total of 40,739 cells were processed, of which 9007 cells were extracted from the control (CON) decidual, 8654 from the air pollution (AP) decidual, 4410 cells from CON junctional, 5665 from AP junctional, 5978 cells from CON labyrinthine and 7025 from the AP labyrinthine layers (Table S1).

**Figure 1:**
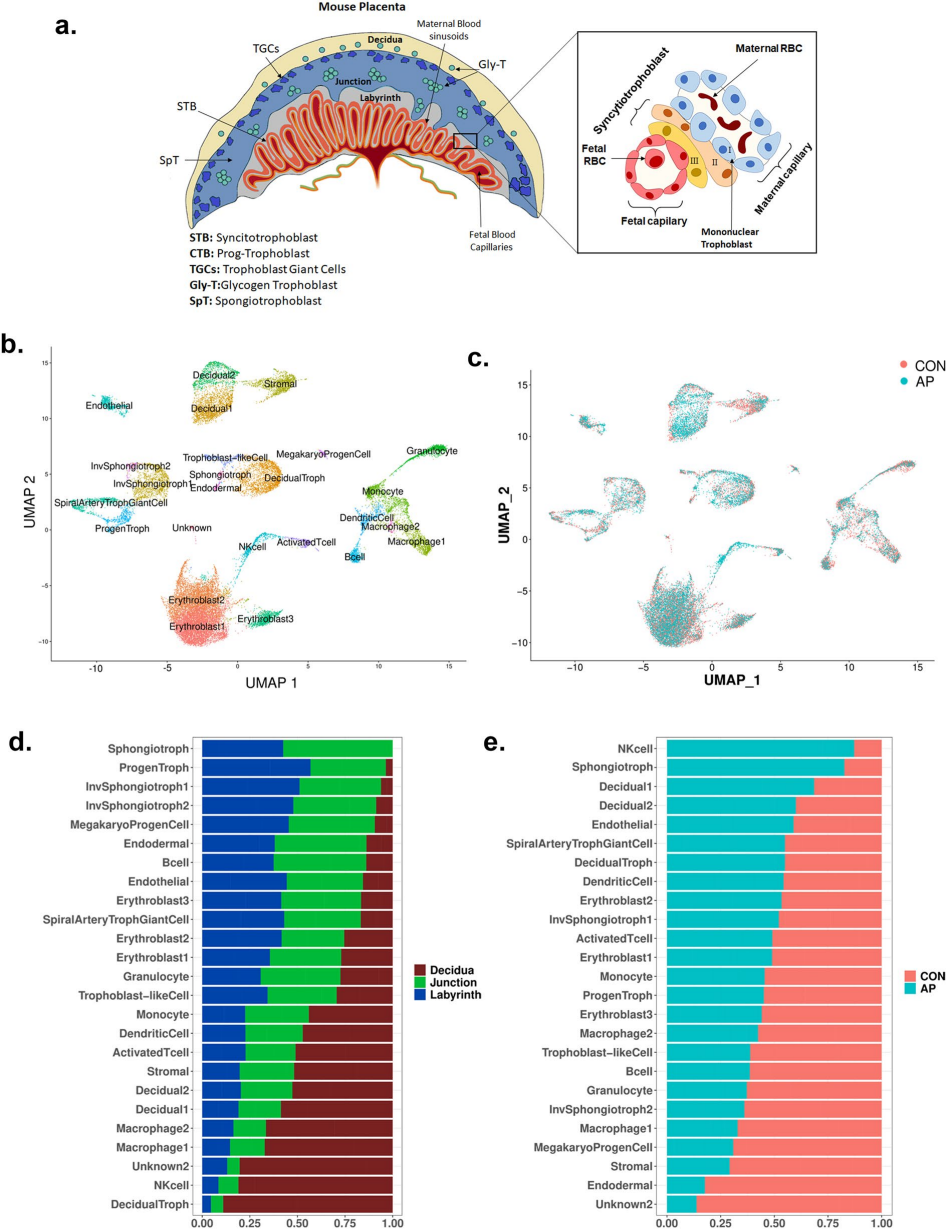
(**a**) Schematic representation of mouse placenta and location of various cell types in different placental compartments. (**b**) Uniform Manifold Approximation and Projection (UMAP) plot of cell and tissue clusters detected in scRNAseq in all 6 samples. Sub-clusters of related cell types could be detected for immune cells, decidual cells, endo/epithelial-like cells, erythroid cells and trophoblasts. (**c**) UMAP projection of cells originating from air pollution (AP) samples (n = 3) and control (CON) samples (n = 3). (**d**, **e**) Quantification of each cell type by placental layer, decidual, junctional and labyrinthine (**d**) or treatment groups, namely AP and CON (**e**). Cell counts were normalized by the total cell count per sample and depicted as fractions of the total cell count for each cell/tissue type.

A schematic of placental cell type(s) distribution is depicted in Fig. 1a. To display various cell clusters of E19 mouse placentas, uniform manifold approximation and projections (UMAP) were applied to the scRNAseq data. Twenty-five distinct clusters were identified, amongst which 24 were annotated to cell types based on signature genes (Fig. 1b) as described in the Mouse Cell Atlas^31^. The majority of these clusters were grouped together based on cell-type similarity, and all cell types could be found in both AP and CON conditions (Fig. 1c). We observed variation in cell type composition among the three different placental compartments, namely the decidual, junctional and labyrinthine (Fig. 1d) regions, and across the two treatment groups (Fig. 1c,e). While the majority of cell types were identified across all placental compartments (Fig. S1), certain cell types were identified to be predominantly present in the decidua (decidual and stromal cells, decidual trophoblasts, macrophages and NK-cells). Other cell types were more abundant in the junctional and labyrinthine layers (B-cells, endothelial and endodermal cells, erythroid cells, invasive spongiotrophoblasts and spongiotrophoblasts) (Fig. 1d). The trophoblast lineage, as the major cell type(s) that shapes the placenta, consists of numerous subtypes present at various stages of differentiation, ranging from undifferentiated progenitor trophoblast to fully differentiated syncytiotrophoblasts. Based on the scRNAseq data we detected an increase in certain structural and proliferating trophoblasts following AP exposure (Fig. 1e). NK-cells, spongiotrophoblasts, and decidual cell-types were enriched upon AP exposure, while other cell types such as granulocytes, macrophages, endodermal and stromal cells were depleted upon AP exposure. However, due to the lack of biological replicates (as all animals were pooled into a single sample to generate sufficient cell numbers) in the scRNAseq analysis, these results are not statistically significant.

### Deconvolution of bulk RNAseq data from whole placentas with scRNAseq as a reference

**Figure 2:**
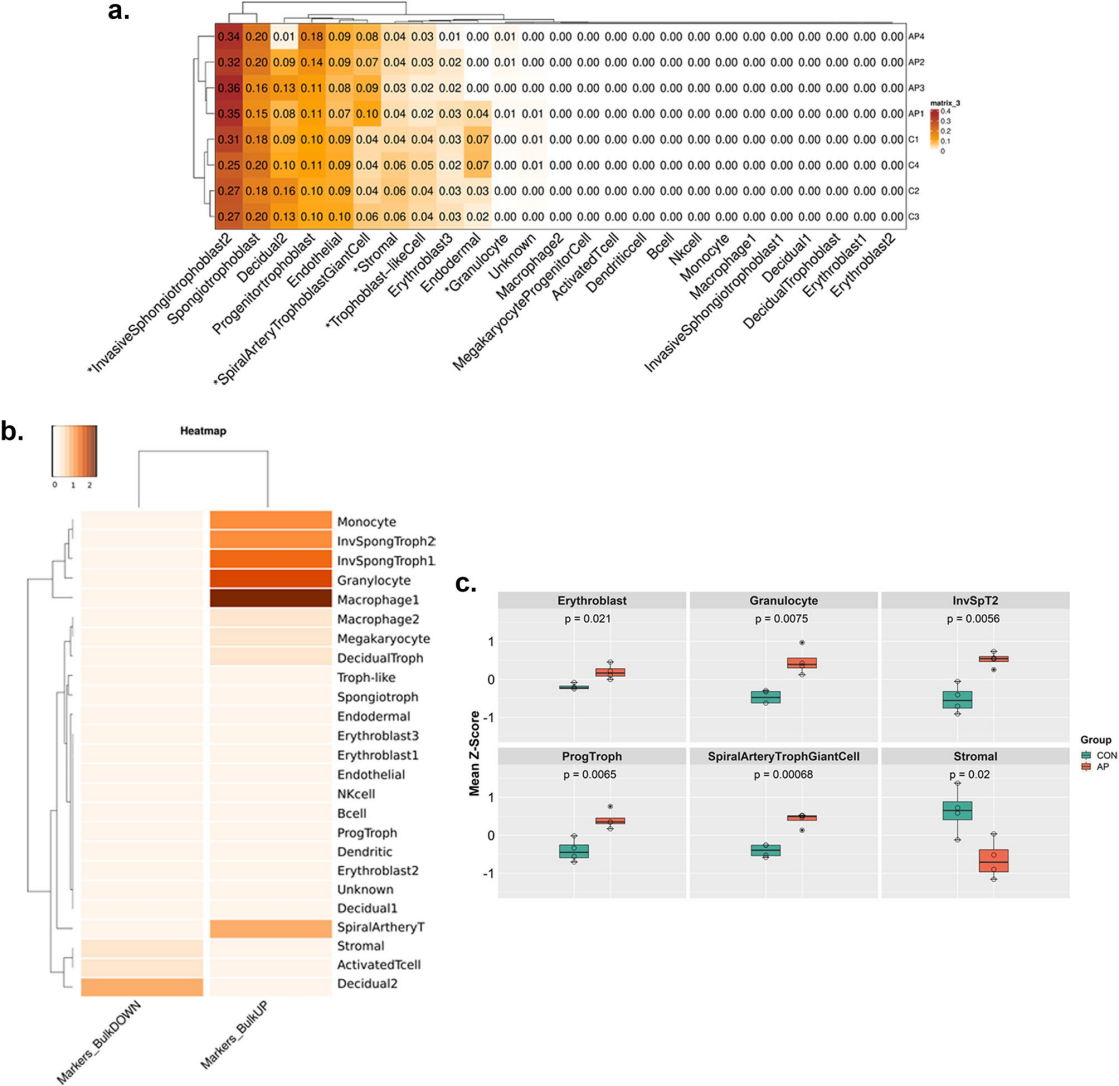
Deconvolution of Bulk RNA-Seq data. (**a**) Cell-type deconvolution of bulk RNAseq data based on pseudobulk scRNAseq as a reference. Numbers present a fraction of the total count. Stars represent an FDR-corrected significant difference (t-test) between the detected cell type fractions in AP (air pollution; n = 4 sequenced samples obtained from 6 pregnant mice) and CON (control; n = 4 sequenced samples obtained from 6 pregnant mice) at a level below 0.05. (**b**) Overlap between the 50 top marker genes for each tissue (Supplementary Table 2) type in the scRNAseq dataset and genes up- or downregulated in AP versus CON detected by bulk RNAseq (Supplementary Table 3). A higher value (depicted with darker color) represents a higher level of overlap. (**c**) Differential abundance of cell types based on z-scores calculated using cell specific marker genes from scRNAseq analysis which were significantly expressed among all samples and filtered by *p* value < 0.05. Students t-test was performed to calculate the significance between two groups InvSpT1 = Invasive Spongiotrophoblasts Type 1. InvSpT2 = Invasive Spongiotrophoblasts Type 2.

To further elucidate the cell-type specific impact of air pollution on placenta, bulk RNAseq data was deconvoluted using gene expression from scRNAseq. To perform an unbiased cell type deconvolution of bulk RNAseq, we created an average pseudo bulk expression matrix from the 25 cell types in the scRNAseq dataset and used this as a reference for deconvolution. We used GEDIT^32^ to perform the deconvolution analysis with the bulk RNAseq data from AP and Control samples as inputs (n = 4 that arose from 6 separate mice, respectively), as GEDIT was shown in a recent report to compare favorably with other deconvolution tools^33^. A second tool, designed specifically for scRNAseq references, MuSic^34^ was also used to verify the deconvolution results. While both tools performed similarly in identifying cell types present in bulk (Fig. 2a and S2a), GEDIT was able to identify a higher number of cell types compared to MuSic. A comparison between the two methods was performed, and the concordance (R^2^ value) as calculated for the CON group was 0.9 and for the AP group was 0.95 (Fig. S2b). Our analysis showed a significantly higher representation of various trophoblast cells i.e. invasive spongiotrophoblast2 (ISpT) and spiral artery trophoblast giant cells along with granulocytes in AP treated samples whereas, stromal cells and trophoblast-like cells showed a significant decrease (Fig. 2a). A similar trend of increased and decreased abundance was observed among other subtypes of spongiotrophoblast and decidual cell subtypes respectively, which did not reach statistical significance (Fig. 2a). After performing differential expression analysis on the bulk RNAseq data, comparing AP and Control samples, we detected an upregulation of some cell-specific signature genes, notably, macrophage, monocyte and granulocyte signature genes, as well as invasive spongiotrophoblast-specific genes in AP (Fig. 2b). Conversely, decidual, stromal and activated T-cell specific signature genes appeared to be downregulated in AP.

To validate the deconvolution results, we generated a z-score normalized expression matrix from bulk RNAseq using cell specific marker genes from our scRNAseq analysis. These markers were considered in the analysis if they had been found as being cell-type specific with a maximum *p* value < 0.05. Although this method only provides relative and not absolute abundance estimates, it can evaluate the relative representation of various cell types in bulk RNAseq. This analysis further revealed a significantly enhanced representation of invasive spongiotrophoblasts and progenitor trophoblast cells among AP samples (Fig. 2c). A relevant increase of spiralarterytrophoblast giant cells was also noted in the AP group. We have also noticed a significant increase in erythroblasts which are the progenitors of red blood cells. Furthermore, we observed a significant reduction in the abundance of stromal cells in AP (Fig. 2c). These cell types depleted in AP are primarily responsible for the construction of the placental vasculature. Hence, an impaired vasculature due to air pollution exposure is apparent. Cytotrophoblasts or progenitor trophoblasts are considered precursors of various invasive trophoblasts or syncytiotrophoblasts. However, these two subpopulations originate from different progenitors with distinct survival characteristics. In our study, we have not observed any alterations in either gene expression pattern or the abundance of these cell types (Fig. S3).

### Immune cell abundance in AP-treated placentas

**Figure 3:**
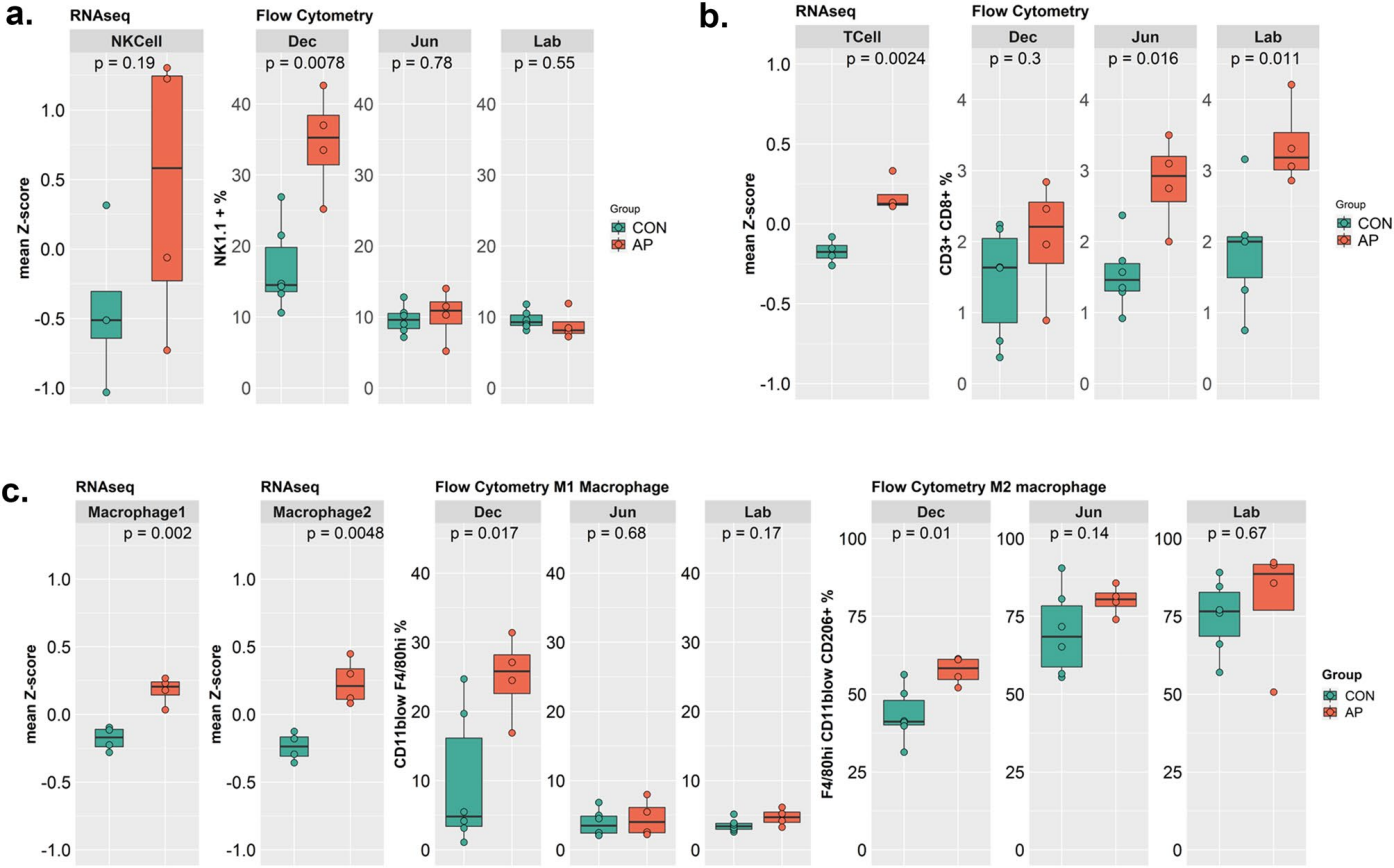
Abundance of various immune cells in different placental compartments: (**a**, **b**) showing the abundance of NK cells (**a**) and T cells (**b**) in placentas of CON or AP group based on Bulk RNA-seq (left panel; n = 4 sequenced from each group obtained from n = 6 pregnant mice) or Flow cytometry data (right panel; n = 6 from each group) from three different compartments of placentas. (**c**) abundance of total macrophages between CON and AP groups (left panel) as obtained from Bulk RNA-Seq and abundance of M1 and M2 macrophages from three different compartments of mouse placentas by Flow cytometry (right panel). Statistical analysis was undertaken using the Student’s t-test to compare AP versus CON groups with significance achieved at a *p* value < 0.05.

Apart from trophoblasts and other structural cells, bulk RNAseq revealed a significant increase in various immune cells including granulocytes, monocytes, B lymphocytes, macrophages and activated T-cells in AP samples compared to controls. Maternal leukocytes of myeloid or lymphoid origin were also enriched in the decidual layer, predominantly among the samples of the AP group. Our analysis of bulk RNAseq revealed an increase in the NK cell population among AP samples which was also supported by the scRNAseq data (Fig. 1e). In addition, data obtained from flow cytometry showed a significant increase in NK cells within the decidual fraction of the AP group (Fig. 3a). We have also identified activated T-cells, predominantly in the decidual layer (Fig. 3b). Using bulk RNAseq showed a significantly higher abundance of activated T-cells in the AP group (Fig. 3b), without any significant difference in expression between AP and CON in the scRNAseq data. Activated T-cells usually secrete pro-inflammatory cytokines that can damage the placental structure, resulting in dysfunction, thereby impairing maintenance of pregnancy. Data obtained from flow cytometry also indicated a significant increase in CD8/cytotoxic T-cells within the junctional and labyrinthine regions of the placenta exposed to AP. A tendency towards an increased T-cell population is also evident in the decidual region of the same group (Fig. 3b) using flow cytometry, however, it was not significant. We also observed small clusters of B-cells and dendritic cells from the scRNAseq analysis, but no differences were evident from bulk RNAseq or flow cytometry data analysis.

Besides NK and T-cells, the other predominant immune cells in the placenta are macrophages which act as primary antigen presenting cells, especially in the decidua during a normal pregnancy. Macrophages are tissue resident immune cells, regularly replenished by circulating monocytes, that exist in two different states of activation, defined as M1 and M2. We have observed higher representation of monocytes and macrophages in the decidual region of placenta and deconvolution of bulk RNAseq data revealed up-regulation of macrophage and monocyte specific genes. Although layer or region-specific location and cell subtype specific information are missing from the bulk RNAseq analysis, the overall representation of various macrophages was increased in AP. We found two distinct clusters of macrophages and both of them were significantly increased in AP samples (Fig. 3c), although these two cell types could not be defined as M1 and M2 macrophages in the current dataset. Moreover, flow cytometry showed significant enrichment of both M1 and M2 macrophages, especially in the decidual layer among the AP samples (Fig. 3c).

### Differentially expressed gene (DEG) analysis from bulk RNAseq data

**Figure 4:**
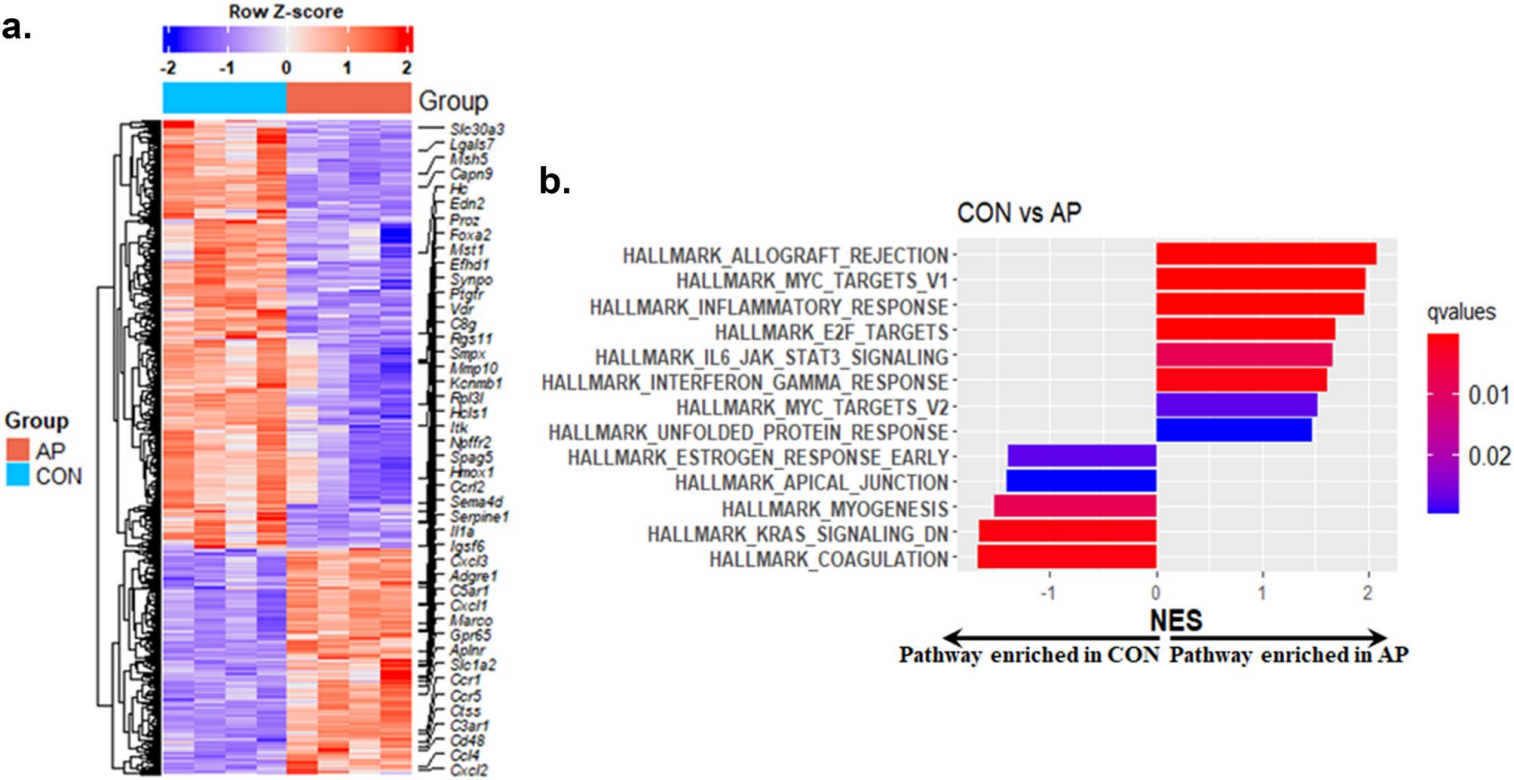
(**a**) Differentially expressed genes (DEGs) obtained from placental samples of control (CON) and air pollution (AP) exposed mice. DEGs were selected based on FDR < 0.05 and Log2Foldchange > 2. Genes which are part of any of the pathways in the right panel have been labeled. A full list of differentially expressed genes can be found in Supplementary Table 3. (**b**) List of Hallmark pathways positively or negatively enriched between AP and CON groups obtained using GSEA are shown.

Next, we set out to analyze the bulk gene expression and identify genes influenced by AP exposure. We observed 118 differentially expressed genes (DEGs) in bulk RNAseq out of which 48 genes were significantly upregulated and 70 genes were downregulated in placentas of mice exposed to AP (Fig. 4a and Supplementary Table 2). We used an FDR value of < 0.05 and a fold difference of > 4 in selecting DEGs to minimize false positive outcomes. Gene Set Enrichment Analysis (GSEA)^35^ using all expressed genes showed positive enrichment of pathways related to inflammation and proliferation typical for lymphocytes and Natural Killer (NK) cells including Allograft Rejection, Inflammatory Response, E2F Targets, Myc-Targets-V1 in the AP group versus CON (Fig. 4b). Conversely, pathways related to signaling in general or development were reduced in AP compared to CON. Pregnancy involves close apposition of two disparate tissues: the uterus and placenta. The tenets of transplantation immunology predict that the placenta along with the fetus would be rejected like all genetically mismatched organ transplants. However, there are mechanisms in place which attenuate immune surveillance of the fetal–maternal interface during gestation by minimizing exposure to maternal T-cells and activation of NK and activated T-cells, thereby producing immune tolerance. In the present study, this attenuation of immune surveillance may be lost in response to AP exposure, setting the stage for pregnancy related adverse outcomes.

## Discussion

A strong association between exposure to particulate matter (PM) pollutants found in traffic related air pollutants, and pregnancy complications such as preterm labor or pre-eclampsia has been reported previously, indicating a pivotal role for placental dysfunction leading to these conditions^6,7,36^. However, these associative studies lack information regarding the cellular mechanisms responsible for such outcomes. Exposure to a low level of PM (namely PM_2.5_) is known to damage trophoblast cells either by direct cellular endocytosis of PM or due to a local oxidative stress or inflammatory response to the particle and other components such as endotoxins or poly aryl hydrocarbons (PAHs), within the pollutants^6,7,12,29,36,37^. The placenta is essential for a successful pregnancy and for maintaining the health of both mother and fetus. The mature placenta is heterogeneous in nature with a panoply of diverse cell types expressing unique transcriptomes^38,39^. However, conventional bulk RNAseq can provide an average expression of signals for an ensemble of cells, without distinguishing between the cell types. We therefore aimed to resolve this issue by adopting scRNAseq using single cell suspensions obtained from three different compartments of the placenta. While scRNAseq is a useful tool for identifying cell-type specific profiles and detecting markers, this technique is usually computationally challenging with a low signal to noise ratio due to the high variability. This method is also not ideal for characterizing the cell type proportion from a solid tissue as the cell dissociation process by itself can introduce bias as larger cells such as the syncytiotrophoblasts may be excluded by the 10X genomic platform^40^. By contrast, bulk RNA-seq measures the average expression of genes from all cell types, an outcome of cell type specific gene expression weighted more by the cell type proportion, and provides a better sequencing depth, thereby increasing the resolution of gene expression analysis. Hence, bulk RNA-seq is more cost-effective when evaluating adequate numbers of biological replicates in order to attain sufficient experimental power. However, unlike scRNAseq, bulk RNA-seq lacks cell- or region-specificity, which could be important to the clinical condition as certain cell types may be more involved than others in a specific response. In our study, scRNAseq data obtained from placenta of AP exposed mice showed damaged trophoblast and placental vasculature along with inflammation. These findings were further validated by estimating the abundance of each placental cell type from bulk transcriptional profiles of heterogeneous samples using signature genes derived from the scRNAseq analysis. Since, neither of these methods are ideal, each having their own limitations, and the fact that certain placental cells, namely trophoblasts, are highly heterogeneous, we used a combined approach utilizing signature genes and cell type abundance inferred by deconvolution of bulk RNAseq. In addition, we used flow cytometry to assess the abundance of immune cells arising from three different placental compartments. We noticed some discrepancies in the cell abundance of macrophages and granulocytes detected by scRNAseq and estimated by bulk RNAseq/Flow cytometry. There can be multiple sources of these discrepancies, such as technical bias or lack of biological replicates in the scRNAseq or inadequate cell-proportion estimation from bulk RNAseq. These limitations highlight the importance of employing multiple methodologies to verify cell abundances when estimating tissue heterogeneity.

Multiple immune cell types including different leukocyte subtypes are recruited into different compartments of the placenta in response to chemokine gradients created by trophoblasts or stromal cells^41–43^. Although these leukocytes are present throughout pregnancy, their abundance changes temporally with a reduction at term. Most of the immune cells in the decidual layer of the placenta are NK cells that regulate vascularization, thereby establishing utero-placental vascular connection and ensuring adequate blood flow from mother to fetus^44,45^. The presence of inflammation during pregnancy abnormally activates NK cells with perforin secretion, which is one of the main mediators of cytotoxicity. Mounting evidence has also linked adverse NK cell activation to reproductive failure in human and mouse^45–52^. In our study, we detected an increased abundance of NK cells in AP exposed placentas which can in turn adversely affect the placental cells that contribute towards maintaining its architecture and thereby normal structure.

We have also observed increased numbers of M1 macrophages in the decidua, and CD8 T-cells in response to AP exposure. M1 macrophages act as antigen presenting cells and skew the T cell response towards TH1 mediated cellular immune response^53^. Accumulation of macrophages, plasma cells and T lymphocytes in the placenta have been reported in response to certain inflammatory conditions, such as chronic decidualitis, villitis of unknown etiology (VUE) and at times even in chronic chorioamnionitis^54^. Of note, T cell accumulation is also observed in solid organ transplant rejection^55^. VUE, is an inflammatory state described to mimic graft rejection, and noted in maternal auto-immune disorders^56–58^, which often complicates a pregnancy and at times leads to preterm labor. Normal pregnancies are characterized by immunological tolerance of the allogenic fetus, which is mediated by the absence or scarce presence of classical MHC class I receptors on placental trophoblasts designed to prevent NK or T cell mediated cytotoxic targeting within the decidua^45,59,60^. In the case of AP exposure, in our present study, we have seen increased numbers of cytotoxic CD8+ T cells not only in the decidua but also in the junctional and labyrinthine regions of the placenta.

GSEA analysis showed positive enrichment of the allograft rejection pathway and immune response pathway genes (predominantly expressed by macrophages or lymphocytes) in placentas exposed to AP. Besides M1 macrophages, there is also an increased abundance of M2 macrophages in the decidual region of AP placentas. Unlike M1, M2 macrophages are immunosuppressive and facilitate tissue remodeling^53^. In parallel, increased abundance of progenitor trophoblasts and various subtypes of invasive spongiotrophoblasts were detected in response to AP exposure. These findings support a compensatory M2 response geared towards reducing the encountered AP induced inflammation whereas actively dividing trophoblasts may target the rebuilding of placental vasculature^61^ or the overall placental architecture. Alternately, the AP induced increase in progenitors may signify a downstream arrest in cellular maturation leading to premature yet terminal differentiation of certain trophoblastic cell types. It is intriguing to apply a similar analysis by deploying insights gained from scRNAseq towards deconvoluting bulk RNA-seq analyses from other conditions known to adversely affect placental vasculature and structural components, as encountered in our previous studies related to maternal caloric restriction induced reduction in uteroplacental blood flow^26^, or in murine models of pre-eclampsia^62^, and in genetically modified mouse models culminating in fetal growth restriction^63^. In addition to the advantage of applying such deconvolution strategies to other murine conditions of clinical significance in the human, the addition of spatial transcriptomics in the future may help decipher the spatial configuration of various identified cell types within their normal habitat within the placenta^64^, shedding additional light on the clinical problem at hand.

We did not examine the timing of birth or other birth outcomes as has previously been reported in rodents by others, where resorption of embryos, preterm birth and reduction in fetal weights emerged, in response to pre-gestational and gestational air pollution exposure^29,65–67^. In addition, while we did not decipher any changes in litter size or placental weights at gestational day 19, similar to previous reports^29,66^. We preliminarily observed cellular necrosis and hemorrhages histologically in placentas exposed to AP versus CON. In addition, we noted that a single dimensional immunohistochemical analysis of immune cells within placental tissue sections using antibodies against specific markers proved insensitive in detecting differences between the two groups, necessitating scRNAseq and flow cytometric analyses as described above.

Although the current study is limited due to pooling of biological replicates into a single sample to meet the requisite cell numbers necessary for scRNAseq, combining scRNAseq and bulk RNAseq along with Flow cytometry provides a good toolbox to characterize the effects of AP exposure on the placenta at the cellular and molecular levels. The cellular changes we have observed could provide the missing link between adverse pregnancy outcomes such as initiation of preterm labor or pre-eclampsia and AP exposure, thereby helping focus the development of preventive strategies for at-risk pregnancies.

## Materials and methods

### Preparation of particulate matter (PM), experimental animals and exposure

The Airborne PM of Standard Reference Material (SRM1649b) was purchased from the National Institute of Standards and Technology (Gaithersburg, MD, USA). Prior to exposure, SRM1649b was re-suspended in sterile saline and sonicated for 15 min to obtain a final concentration of 15 μg/µL, and ensuring uniform suspension of dissoluble PAHs, PCBs, and inorganic constituents with a heterogeneous PM size reduced to a range between ~ PM_2.5_ and ~ PM_10_, thereby mimicking traffic-related air pollutants. C57BL/6J mice obtained from Jackson Laboratories were housed in filter-topped cages under dark:light 12:12(h) cycling conditions with ad lib access to water and chow diet (TD. 06414, Harlan Teklad Laboratories, Indianapolis, IN, USA) within the University of California Los Angeles Animal Care Facility. Female mice were administered SRM1649b intranasally under light restraint, beginning from 2 months prior to gestation until 18th day of gestation (G18; term being ~ G21) every third day or daily during gestation only (G1–G18). The experimental group received 20 μL (10 μL/nares) of SRM1649b (300 μg/day/mouse) suspension while the control group received 20 μL of sterile saline^68^. Placentas were collected at gestational d19, following laparotomy and hysterectomy, under 4% for induction and 1.25 to 1.5% for maintenance of the inhalational isoflurane anesthesia. Protocols for the care and use of mice were approved by the Animal Research Committee of the University of California following the guidelines provided by the National Institutes of Health. The study was carried out in compliance with the ARRIVE guidelines^69^.

### RNA extraction and library preparation for bulk RNAseq

RNA was extracted with Direct Zol RNA MiniPrep kit from Zymo (R2050, USA) using manufacturer’s instructions. RNA sequencing libraries were prepared using KAPA Stranded mRNA sequencing kit (# KK8420; Kapa Biosystem, Cape Town, South Africa) following manufacturer’s protocol. Briefly, 100 ng of total RNA from each sample was used as the starting material with biological quadruplicates from each group. mRNA was captured using magnetic oligo-dT, fragmented by heat and Mg, and reverse transcribed to cDNA using random primers. After 2nd strand synthesis, cDNA was end-repaired, index adapter-ligated and PCR amplified. SPRIselect beads (# B23318, Beckman Coulter, Indianapolis, IN, USA) were used to purify nucleic acids after each step of the library preparation. RNA sequencing libraries were sequenced by the HiSeq-4000 sequencer (Illumina Inc.; San Diego, CA, USA).

### Bulk RNAseq analysis

Raw sequences were demultiplexed using a custom script. The demultiplexed fastq files are available at GEO (GSE178233). Quality of the raw fastq files was reviewed using FastQC^70^. Single end reads from individual libraries were aligned to the mouse reference genome (mm10) using STAR-2.6 aligner^71^. From each library, uniquely aligned reads were used to obtain raw counts for all the *Mus musculus* genes (mm10; ucsc genome annotation) with the featureCounts tools from the Subread package^72^. Further analysis and data visualization were performed using various R packages. DESEq2^73^ was used to calculate the size factors and Raw read counts were scaled accordingly to normalize for library size. Low abundant genes were filtered out at this point. Differential expression was calculated between control and AP samples using a negative binomial generalized linear model, and correction for multiple testing was represented as FDRs (false discovery rates) using the Benjamini–Hochberg method. FDR values < 0.05 were considered statistically significant.

### Preparing placental cells for scRNAseq and flow cytometry

Placentas collected at G19 were extensively washed with cold PBS twice and the three different layers (decidual, junctional and labyrinthine) were separated. The whitish gray decidual layer was gently peeled off from the rest of the placenta while maintaining tissue integrity. Junctional zone separation from the labyrinth was done microscopically with a sterile blade. Decidual, junctional and labyrinthine layers from multiple placentas were pooled to obtain adequately optimal numbers of cells for analysis. Separated layers were minced into small pieces and digested with an enzyme cocktail [collagenase (1 mg/ml) (SCR-103, Sigma, USA) and DNase (20 µg/ml) (D-5025, Sigma, USA)] in RPMI 1640 (Thermo Fisher, Cat # 61870036)] for 60 min on a shaker. Single cell suspensions were prepared by passing through 100 μm cell strainers (MACS, Smart Steiner) and numbers of live cells were determined by trypan blue (T8154 Sigma, USA) after lysing erythrocytes with ACK Lysis buffer (Lonza, Walkerville, MD USA) for 3 min at room temperature. These freshly isolated single cell suspensions were directly subjected to scRNAseq using the Chromium Single Cell 3′-Library & Gel Bead Kit (10X Genomics; Pleasanton, CA, USA). For flow cytometry, cells were incubated with pre-optimized concentrations of fluorescence tagged antibodies marking different cell lineages. A list of antibodies used for flow cytometry has been incorporated in the Supplementary Table 4. Doublets were excluded based on forward scatter. At least 150,000 lymphocyte-gated cells were collected for each sample and negative biological populations for each subpopulation were used as references to set up the cut-off values. Data were collected using Fortessa II flow cytometer (BD Biosciences) and analyzed by the FlowJo software (v10.4.0 Tree Star; BD Life Sciences) https://www.flowjo.com/.

### Placental single cell transcriptomics

Library construction: Single cell RNAseq (scRNAseq) libraries were prepared using the Chromium Single Cell 3′-Library & Gel Bead Kit (P/N 1000075, 10x Genomics) following manufacturer’s instructions. Briefly, placental single cell suspension was prepared as mentioned above and loaded onto a Chromium Single Cell Controller to generate single cell gel beads in emulsion (GEM). GEM-Reverse transcription (RT) was performed, and single stranded DNA was cleaned with Dyna beads. cDNA was then fragmented, end-repaired, and A-tailed. Furthermore, adaptor ligation was performed using the Chromium Single Cell 3′-kit followed by post-ligation cleanup using the SPRIselect Reagent.

### Single cell RNAseq (scRNAseq) analysis

The raw fastq files were processed with Cell Ranger v4.0 (10X Genomics) using the cellranger count and cellranger aggr pipelines against the mm10 reference package provided. Seurat v3.0^74,75^ was used for quality control, analysis, and exploration of the data using a standard pipeline. Cell clusters were annotated using the Mouse Cell Atlas v.2.0 marker genes as a guide^31^.

### Cell type identification and signature genes quantification by bulk RNAseq

Deconvolution of bulk RNA-seq was executed using GEDIT v1.6^32^ using a reference matrix from averaged “pseudo-bulk” single-cell RNA-seq data, using the default parameters. Further verification of the results obtained by GEDIT was conducted using MuSiC^21^. For z-score calculation, log normalized read counts obtained from DESEq2 analysis and Z-scores were calculated by subtracting the average read counts (for a single gene) from the read counts of each sample and then dividing the result by the SD of all the read counts following the formula:

$$Zscore=\frac{\left(readcountG-meanreadcountG1...Gn\right)}{SD}\left(G1...Gn\right)$$

SD: standard deviation; G_1_: 1st sample; G_n_: nth sample where n is the total number of samples.

To obtain aggregated Z-scores for each cell type, an average of z-scores from the top 10 signature genes for individual scRNA cell-types were calculated for each sample. Signature genes for cell types missing in our *scRNA* data were obtained from previous reports^21,76^.

### Statistical analysis and data visualization

Unpaired T tests were performed to compare differences in means between CON and AP groups. Any exceptions have been indicated in the text. Data visualization was performed using R^77^ with various packages like Seurat^75^, ggplot2^78^, clusterprofiler^79^, ComplexHeatmap^80^ and final figures were combined using Adobe Photoshop CS6 (2012).

## Supplementary Information

Supplementary Information.
