# Supplementary material for: Integrated analysis of an in vivo model of intra-nasal exposure to instilled air pollutants reveals cell-type specific responses in the placenta
Source: Sci Rep. 2022 May 19;12:8438. doi: 10.1038/s41598-022-12340-z (PMC9119931; doi:10.1038/s41598-022-12340-z)
Supplement: Supplementary file 1 — Supplementary Information. [file 41598_2022_12340_MOESM1_ESM.docx]

**Supplementary materials for: Integrated analysis of an in-vivo model of intra-nasal exposure to instilled air pollutants reveals cell-type specific responses in the placenta**

Anela Tosevska1#†, Shubhamoy Ghosh2†, Amit Ganguly2†, Monica Cappelletti2, Suhas G. Kallapur2, Matteo Pellegrini1* and Sherin U. Devaskar2*


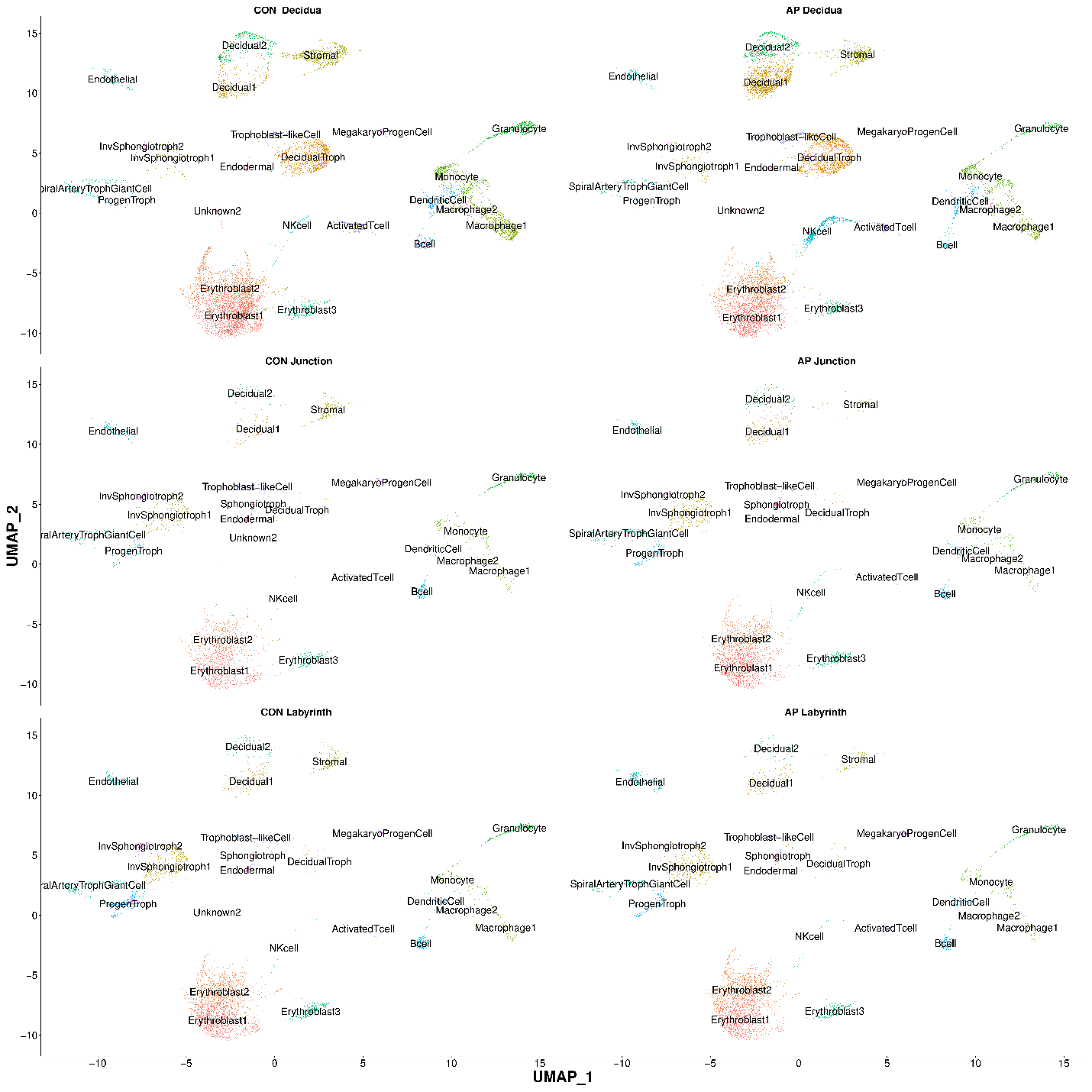


**Supplementary Figure 1.** Uniform Manifold Approximation and Projection (UMAP) plot of cell and tissue clusters detected in scRNAseq in different placental compartments of Control (CON) and Air Pollution material administered samples (AP).


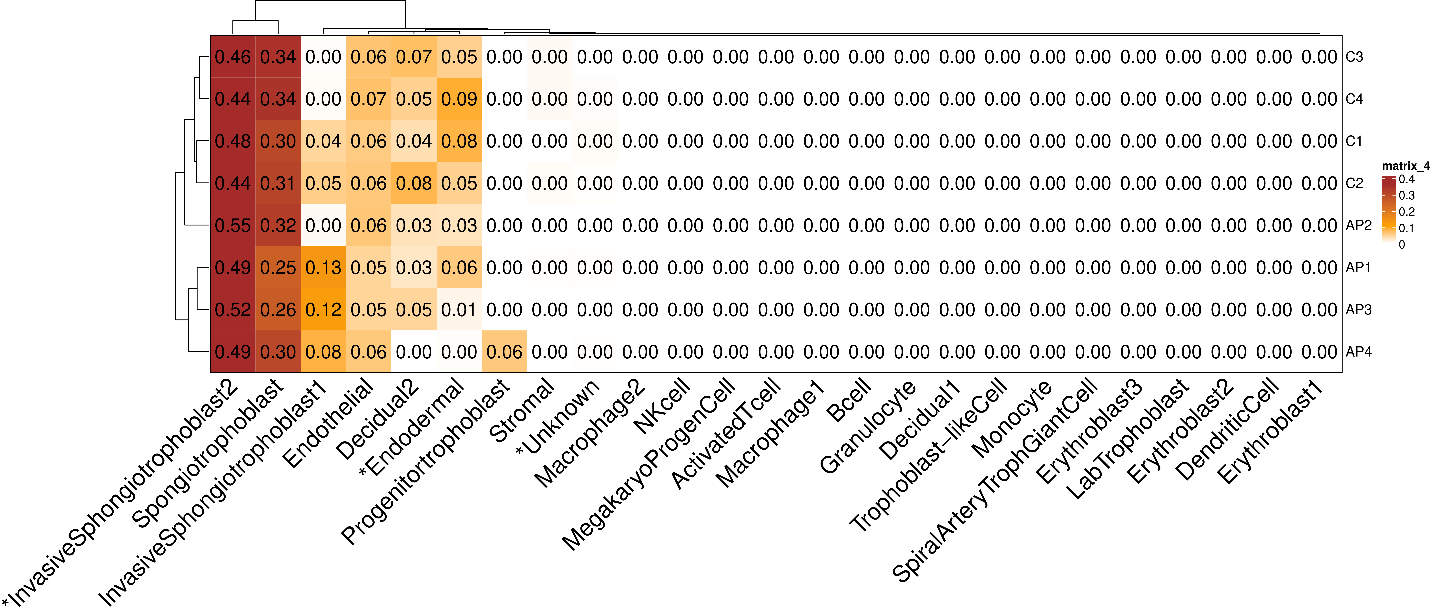


**A**


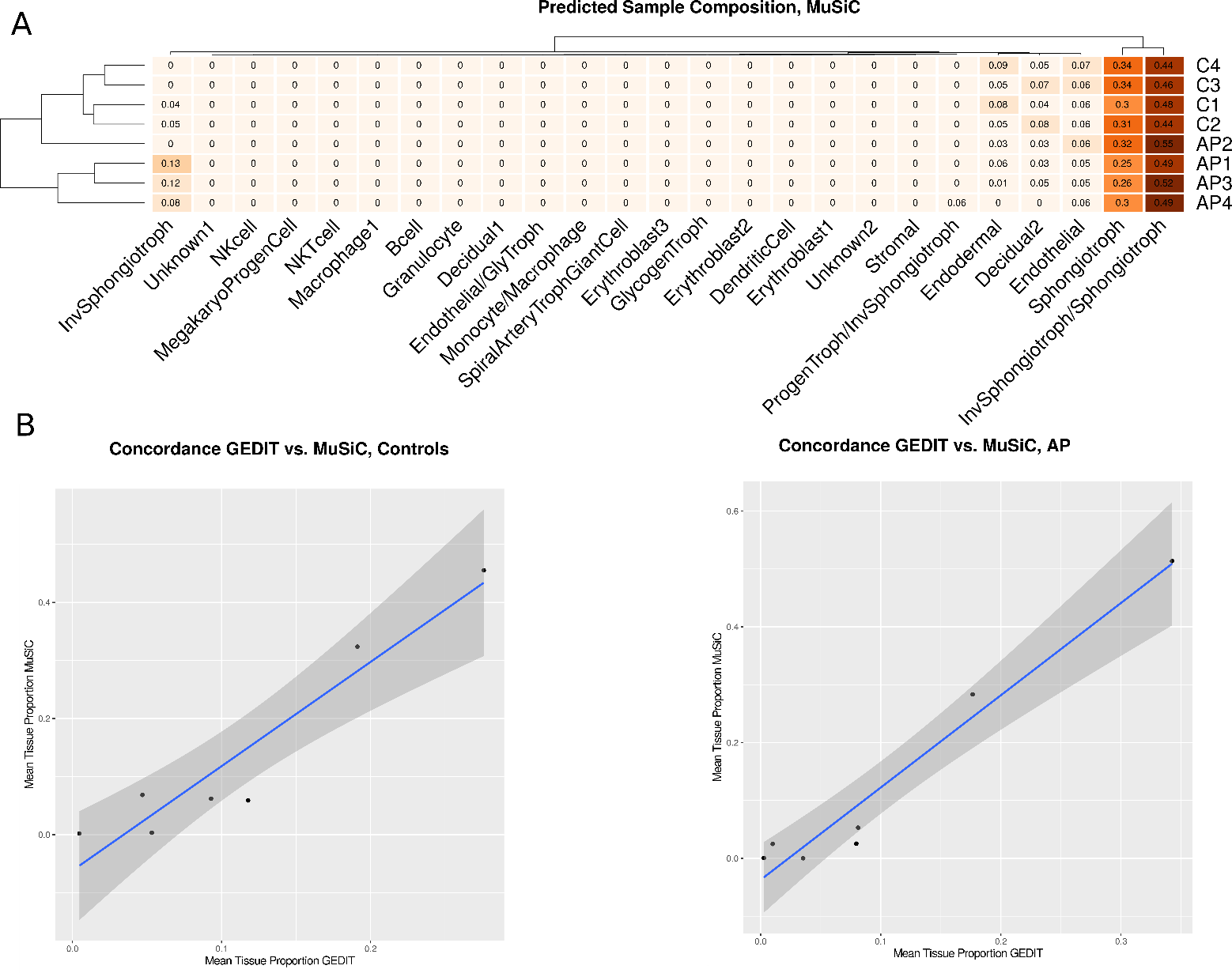


**B**

**Supplementary Figure 2.** Deconvolution of Bulk RNA-Seq data**. A** Cell-type deconvolution of bulk RNAseq data based on pseudobulk scRNAseq as a reference, using MuSiC. Numbers present fractions of the total count. **B** Concordance in the predicted cell-type proportions between GEDIT and MuSiC in CON. R2 = 0.9 (right panel); concordance in the predicted cell-type proportions between GEDIT and MuSiC in AP, R2 = 0.95 (left panel).


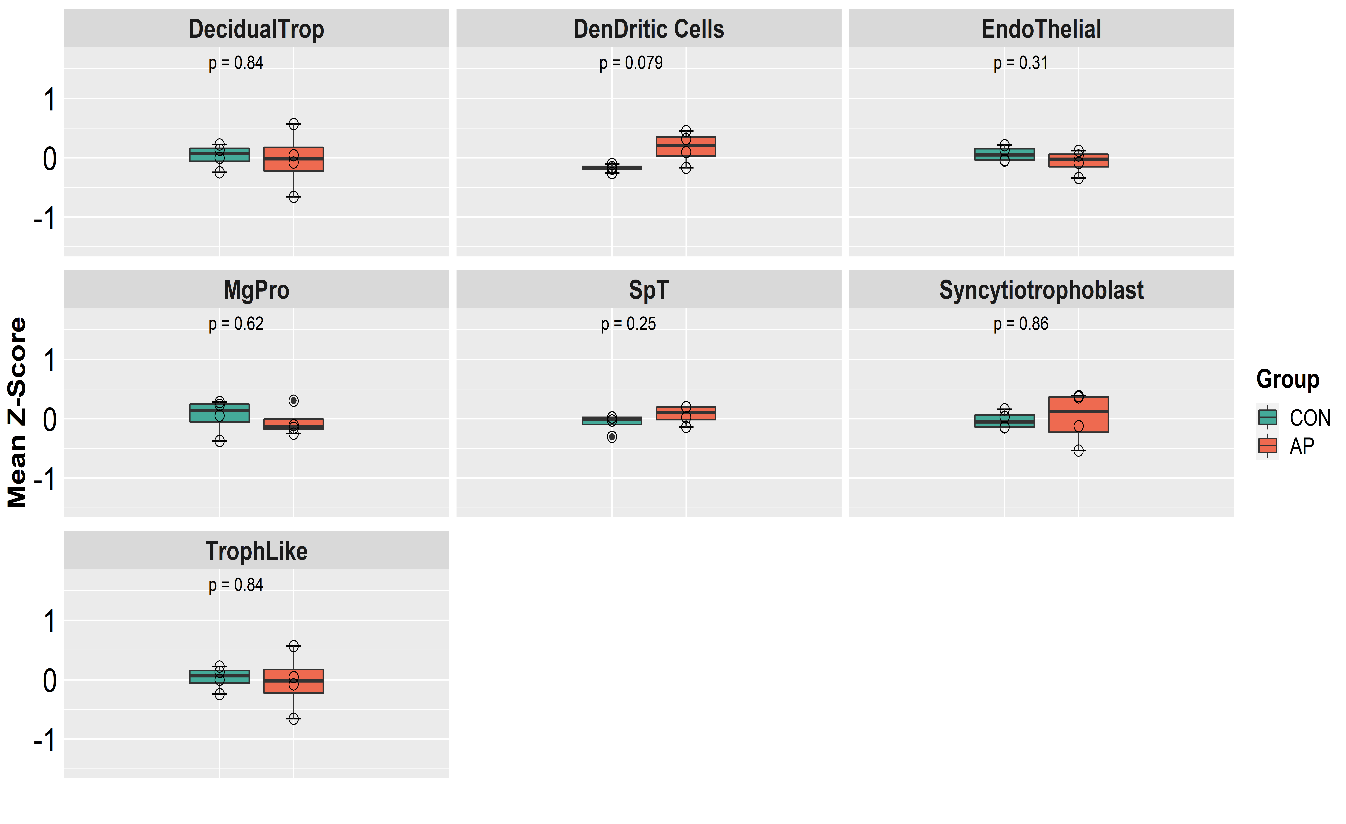


**Supplementary Figure 3.** Differential abundance of cell types based on z-scores calculated using cell specific marker genes from scRNAseq analysis which were significantly expressed among all samples and filtered by p-value < 0.05. Students t-test were performed to calculate the significance between two groups.

**SpT**=Spongiotrophoblast

**MgPro**=megakaryocyte Progenitors.

**TrophLike**= Trophoblast like cells

**GO term**


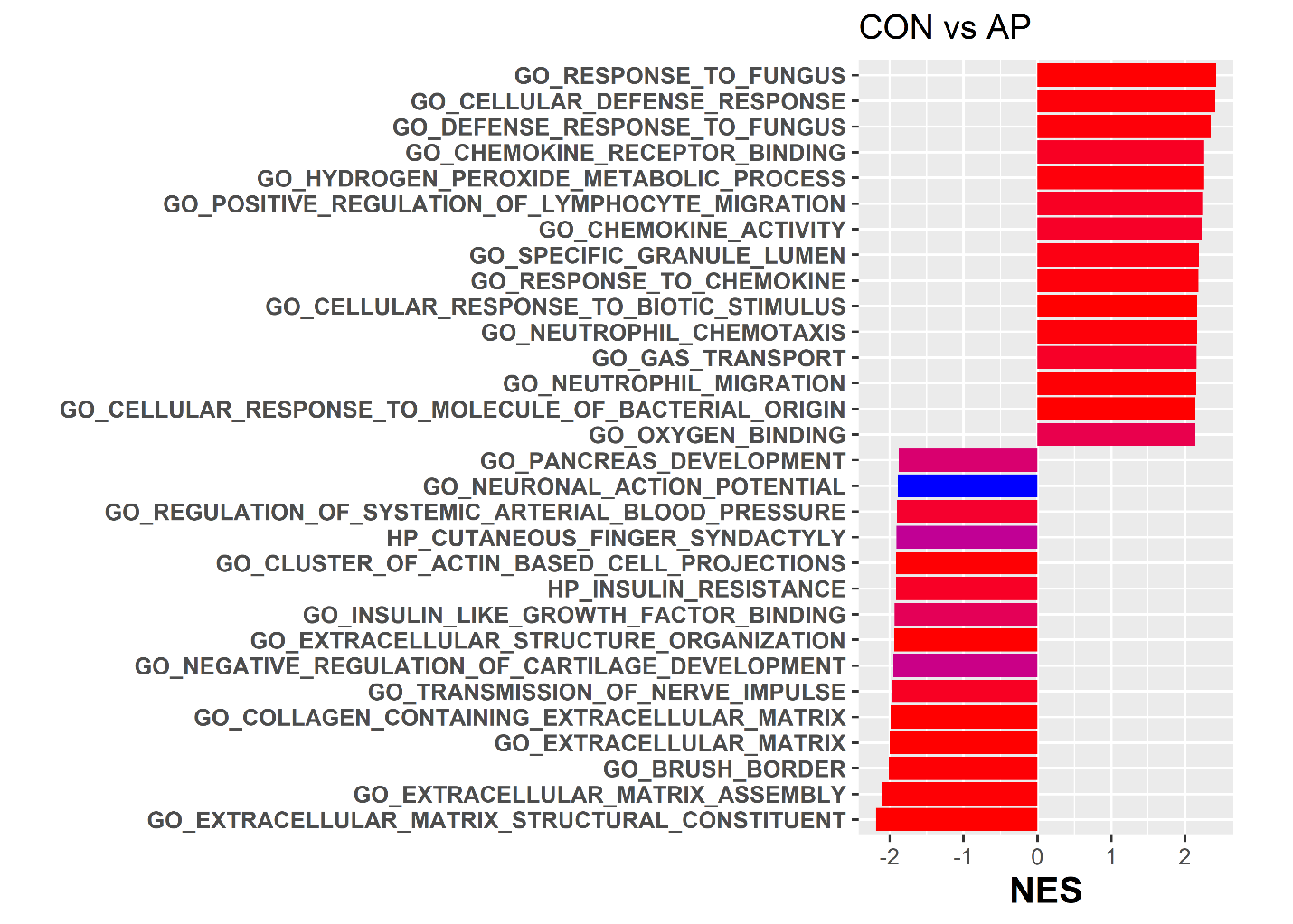


**Pathway enriched in CON**

**Pathway enriched in AP**

**Supplementary Figure 4A.** List of GO Terms enriched in either CON or AP obtained by Gene Set Enrichment Analysis (GSEA). NES represents Normalized Enrichment Score in AP vs. CON.

**Reactome and other Pathways**


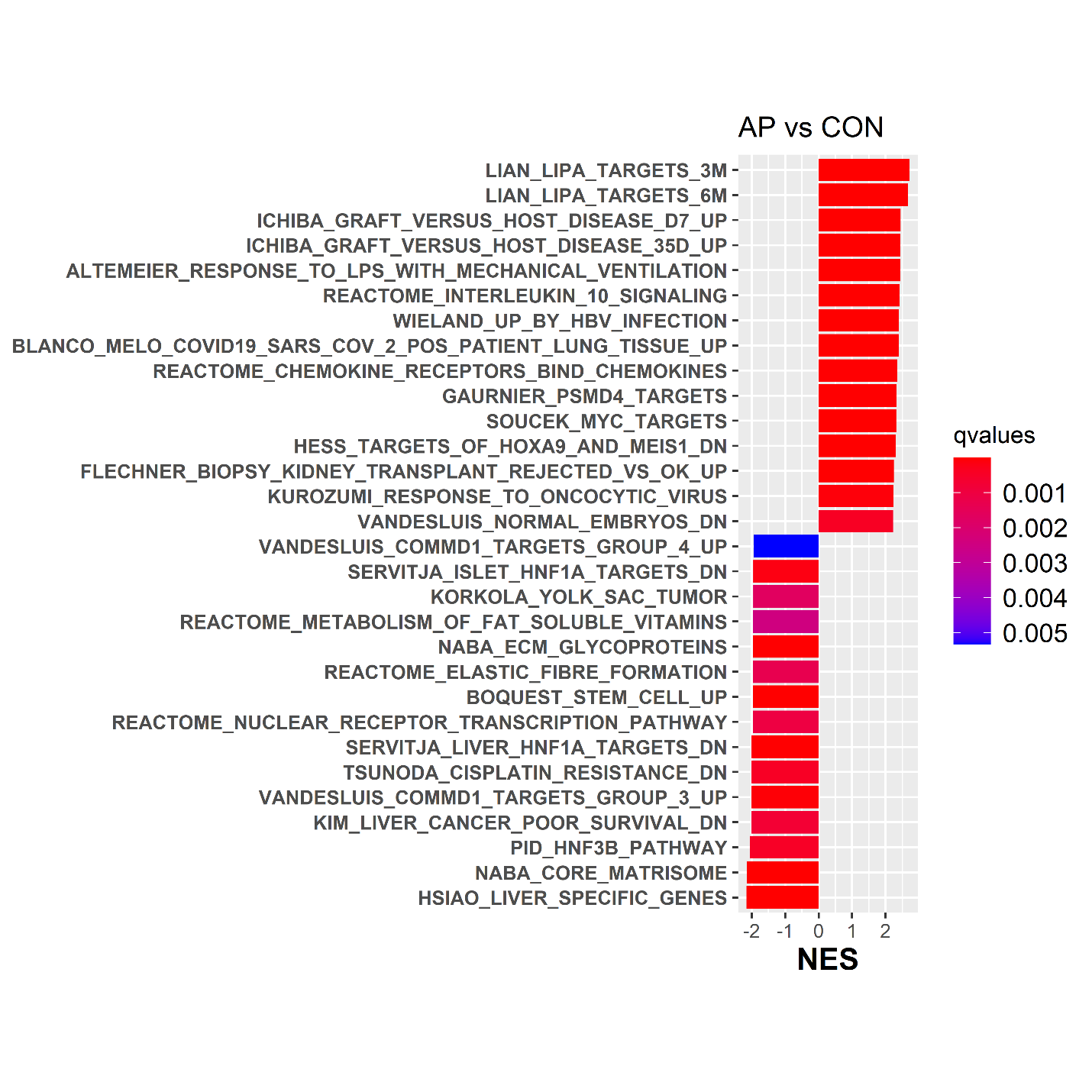


**Pathway enriched in AP**

**Pathway enriched in CON**

**Supplementary Figure 4B**. List of Reactome and other pathways enriched in either CON or AP, obtained by GSEA. NES represents Normalized Enrichment Score in AP vs. CON.


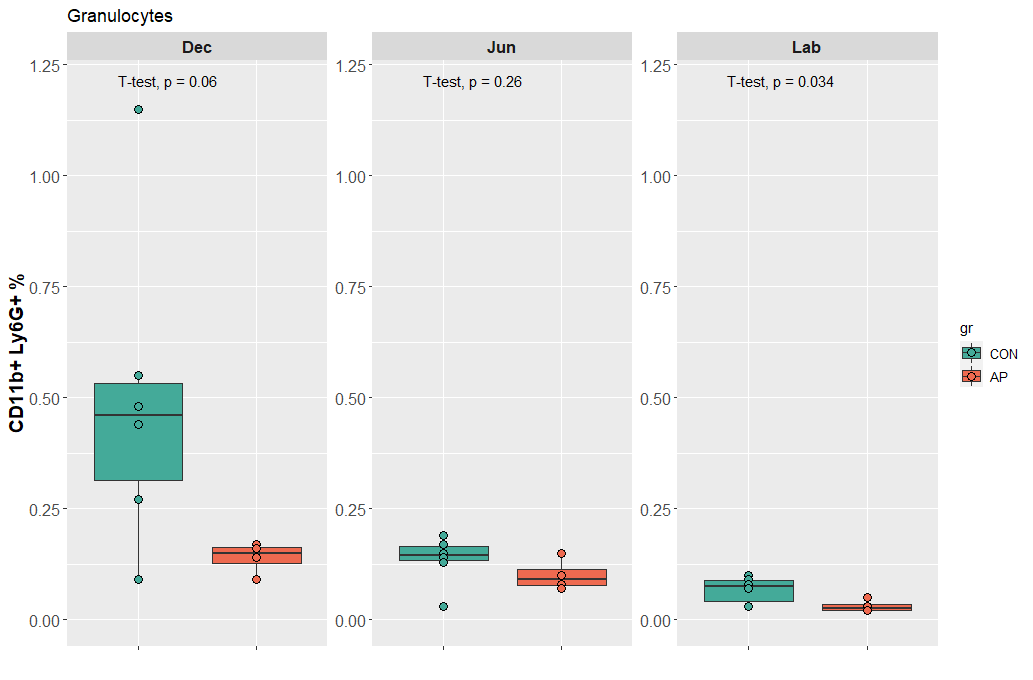


**Supplementary Figure 5**: Abundance of granulocytes in placenta of CON or AP based on Flow cytometry data.

**Supplementary Table 1:** Quality control metrics for scRNAseq.

| Pre-Normalization Total Number of Reads | 855780368 |
| --- | --- |
| Post-Normalization Total Number of Reads | 665351099 |
| Pre-Normalization Mean Reads per Cell | 21006 |
| Post-Normalization Mean Reads per Cell | 16332 |
| Fraction of Reads Kept (CON DECIDUA) | 100.00% |
| Fraction of Reads Kept (AP DECIDUA) | 94.80% |
| Fraction of Reads Kept (CON JUNCTION) | 55.10% |
| Fraction of Reads Kept (AP JUNCTION) | 70.70% |
| Fraction of Reads Kept (CON LABYRINTH) | 75.80% |
| Fraction of Reads Kept (AP LABYRINTH) | 71.30% |
| Pre-Normalization Total Reads per Cell (CON DECIDUA) | 15321 |
| Pre-Normalization Total Reads per Cell (AP DECIDUA) | 15301 |
| Pre-Normalization Total Reads per Cell (CON JUNCTION) | 29481 |
| Pre-Normalization Total Reads per Cell (AP JUNCTION) | 27086 |
| Pre-Normalization Total Reads per Cell (CON LABYRINTH) | 23321 |
| Pre-Normalization Total Reads per Cell (AP LABYRINTH) | 23132 |
| Pre-Normalization Confidently Mapped Barcoded Reads per Cell (CON DECIDUA) | 7402 |
| Pre-Normalization Confidently Mapped Barcoded Reads per Cell (AP DECIDUA) | 7807 |
| Pre-Normalization Confidently Mapped Barcoded Reads per Cell (CON JUNCTION) | 13425 |
| Pre-Normalization Confidently Mapped Barcoded Reads per Cell (AP JUNCTION) | 10463 |
| Pre-Normalization Confidently Mapped Barcoded Reads per Cell (CON LABYRINTH) | 9760 |
| Pre-Normalization Confidently Mapped Barcoded Reads per Cell (AP LABYRINTH) | 10384 |
| Num Cells (CON DECIDUA) | 9007 |
| Num Cells (AP DECIDUA) | 8654 |
| Num Cells (CON JUNCTION) | 4410 |
| Num Cells (AP JUNCTION) | 5665 |
| Num Cells (CON LABYRINTH) | 5978 |
| Num Cells (AP LABYRINTH) | 7025 |
| Cells Total | 40739 |

**Supplementary Table 2:** Top 50 marker genes for each tissue type in the scRNAseq dataset.

| **Erythroblast1** | **Erythroblast2** | **SpiralArtery Trophoblast-Giant Cell** | **Endothelial Cell** | **NK Cell** | **B Cell** | **Erythroid3** | **Progenitor-Trophoblast** | **Dendritic Cell** | **Trophoblast-like Cells** | **NKT Cell** | **Megakaryocyte-Progenitor Cell** |
| --- | --- | --- | --- | --- | --- | --- | --- | --- | --- | --- | --- |
| Hbb-bs | Mki67 | Sult1e1 | Plvap | Gzmc | Igkc | Hba-a2 | 3830417A13Rik | Cd209a | Fxyd3 | Trbc2 | Treml1 |
| Hbb-bt | Slc4a1 | Prl7c1 | Emcn | Gzme | Cd79a | Hba-a1 | Prl2c3 | Ccr2 | Rhpn2 | Cd3g | Gp9 |
| Snca | Snca | Aldh1a3 | Adgrl4 | Gzmf | H2-Eb1 | Alas2 | Cited1 | Klrd1 | Atp1b1 | Skap1 | Gp6 |
| Alas2 | Gypa | Rgs5 | Clec14a | Gzmd | H2-Aa | Hbb-bs | Ghrh | H2-DMb1 | Lmo7 | Cd3d | Alox12 |
| Hba-a1 | Mkrn1 | Cyp17a1 | Tmem100 | Gzmg | Ighm | Snca | Nup62cl | Upb1 | Lsr | Nkg7 | Ppbp |
| Hba-a2 | Bpgm | 1500009L16Rik | Gpihbp1 | Tmsb4x | Ebf1 | Hbb-bt | Fnd3c2 | H2-DMa | Cdh1 | Ms4a4b | Itga2b |
| Bpgm | Hbb-bt | Tmc5 | Ptprb | Anxa2 | Ly6d | Tmsb4x | Ctsr | Plbd1 | Adamts9 | Lat | P2rx1 |
| Ube2l6 | Fam46c | Cdx2 | Egfl7 | Mt1 | Iglc2 | Bpgm | Prl2c5 | H2-Aa | Fnbp1l | Ctsw | Ly6g6f |
| mt-Co2 | Epb41 | Serpinb9d | Cldn5 | Hilpda | Cd79b | Fau | Tfpi | H2-Ab1 | Serinc2 | Cd3e | Gp5 |
| mt-Atp6 | Fech | Spata21 | Clec1a | Rpl32 | Fcmr | H2-D1 | Rhox6 | H2-Eb1 | Dsp | Trbc1 | Gstt1 |
| mt-Co3 | Alas2 | Rgs17 | Tie1 | Sh3bgrl3 | Ighd | Eef1a1 | Ctsj | H2-Oa | Sel1l3 | Lck | Mpig6b |
| Actg1 | Hba-a1 | Mmp15 | Ecscr | Lgals3 | Ms4a1 | Rps27a | Rhox9 | Cd7 | Elf3 | Trac | Mfsd2b |
| Mkrn1 | Hba-a2 | Plac1 | Cyyr1 | Rpl26 | Cd74 | Rpl23 | Krt19 | Cd74 | B3gnt3 | Itk | Clec1b |
| Tmsb4x | Tmsb4x | Serpinb9c | Igfbp3 | Rpl22l1 | Iglc3 | Actb | Bex1 | Rnase6 | Cobl | Cd28 | Myl9 |
| Actb | Trim10 | Prl2c5 | Pecam1 | Rpl36 | H2-Ab1 | Rps8 | Krt18 | Cd83 | Krtcap3 | Icos | Nrgn |
| Fau | Fam220a | Serpinb9e | She | Arhgdib | Gm8369 | Rps3a1 | Prl7d1 | Gpr132 | Clcn2 | Sh2d2a | Vwf |
| Eef1a1 | Ube2l6 | Trap1a | Robo4 | Rpl28 | Bank1 | Mkrn1 | Tpm2 | Klrk1 | Tmc4 | Tnfrsf18 | Gchfr |
| H3f3b | Eef1a1 | Fam89a | Rasip1 | Rpl27 | Mzb1 | Rpl27a | Krt8 | Cytip | Tmprss2 | Gimap3 | Pf4 |
| Hspa8 | Slc25a37 | Prl2a1 | Ushbp1 | Rpl38 | H2-DMb2 | Rpl30 | Hspb1 | Lmo1 | Klf5 | Ltb | Cd226 |
| Ptma | Hbb-bs | Rps6ka6 | Slc9a3r2 | Rpl19 | Pax5 | Rpl37a | Serpinb9g | Tnip3 | Spns2 | Ptprcap | Capn3 |
| Rps27a | Actg1 | Tfap2c | Sox18 | S100a11 | Gm31243 | Actg1 | Abhd6 | Ms4a4c | Eps8l2 | Cd2 | F2rl2 |
| Rps27 | Rpl23 | Ada | Jag2 | Fau | Cd19 | Rpl13 | Plet1 | Clec10a | Ptprf | Il2rb | Rbpms2 |
| Rpl30 | Prdx2 | Prl7d1 | Arhgef15 | Rpl21 | Pou2af1 | Rpl9 | Trap1a | Pmaip1 | Pcbd1 | Gimap4 | Mmrn1 |
| Rps8 | Rpl19 | Dusp9 | Ccm2l | Cd63 | Ccr7 | Rps27 | Sct | Ctss | Rnf128 | Bcl11b | Rgs10 |
| Ppia | Rps20 | Nxf7 | Mcam | Ctsl | H2-Ob | H2-K1 | Prl3b1 | Ciita | Emp2 | Gimap1 | Slc6a4 |
| Rpl23 | Rpl27a | Pappa2 | Dll4 | Gpx3 | Fcer2a | Rps11 | Dusp9 | Ly86 | Myo6 | Il7r | Clu |
| Rps13 | H2-D1 | Rhox5 | Adcy4 | Rpl3 | Vpreb3 | B2m | Crip2 | Ms4a6c | Wfs1 | Gimap6 | Gng11 |
| Rpl9 | H2-K1 | Pla2g4d | Shank3 | Serpinb6a | Iglc1 | Rps16 | Fdx1 | Cbfa2t3 | Cldn7 | Cd247 | Fermt3 |
| Rps16 | Fau | Serpinb9g | Apold1 | Rpl22 | Tnfrsf13c | Rpl35a | Adgrf5 | Lsp1 | Echdc2 | Cd8b1 | Rasgrp2 |
| H2-K1 | Rpl30 | 1600025M17Rik | Mmrn2 | Ifitm3 | Gimap6 | Rps9 | Dsc2 | Fgd2 | Itga6 | Thy1 | Tmem40 |
| H2-D1 | Rps8 | Rufy4 | Stc1 | Ctsz | Cd37 | Rpl32 | Tmem37 | Irf5 | Tspan8 | Txk | Aldh2 |
| Rps11 | Rpl13 | Bex1 | Palmd | Vim | H2-Oa | Rpl18 | Slc38a4 | Alox5ap | Arhgef5 | Klrd1 | Parvb |
| Rpl18 | Rpl18 | Fthl17a | Grrp1 | Psap | Mef2c | Rpl37 | Peg10 | Ppfia4 | Ddr1 | Il18r1 | Thbs1 |
| Rps20 | Blvrb | Psg29 | Rapgef5 | Timm17a | Siglecg | Rps13 | Rhox5 | Flt3 | 2200002D01Rik | Tcf7 | Lyz2 |
| Rps3a1 | Ube2c | Tnfrsf9 | Fgd5 | Gng12 | Satb1 | Rpl19 | Krt7 | Cd52 | Aqp8 | Vps37b | Lat |
| Rpl27a | Rps11 | Irs3 | Ldb2 | Ssr4 | Ptprcap | H3f3b | Serpinb9e | Csf2rb | Zbtb10 | Cd7 | Plek |
| B2m | Rpl32 | Pcdh12 | S1pr1 | Sec11c | Blk | Rps20 | H19 | Itgb7 | Bace2 | Sh2d1a | Samd14 |
| Rps12 | Rplp1 | Prl7b1 | Myct1 | Cd9 | Cd2 | Rpl8 | Sin3b | Ifitm6 | Cldn4 | Stat4 | Cd9 |
| Rps18 | Rpl9 | Fbxo27 | Adgrf5 | Grn | Spib | Rpl26 | Klhl13 | Cd300a | Me1 | Klk8 | Hist1h1c |
| Rps19 | Tpt1 | Gm40975 | Mest | Tpm4 | Ets1 | Rps10 | Hmgn1 | Coro1a | Tinagl1 | AW112010 | Fcer1g |
| Rplp1 | Rpl26 | Phactr1 | Cavin2 | H3f3a | Ltb | Rplp1 | Uaca | Spi1 | Atp9a | Hcst | Pttg1ip |
| Rpl35a | Pnpo | Prex2 | Fam167b | Hmgn1 | Fcrla | Rps3 | Tbrg1 | Traf1 | Ptpn14 | Prkcq | Bin1 |
| Rpl37 | Gpx1 | Peg10 | Esam | Cebpb | P2ry10 | Rpl11 | Eps8l2 | Map4k1 | Flnb | Sept1 | Zyx |
| Rpl26 | Rps27a | Pramef12 | Podxl | App | Foxp1 | Rps19 | Mbnl3 | Gpr171 | Jup | S1pr4 | Cavin2 |
| Rps4x | Ube2o | Cdkn1c | 8430408G22Rik | Sparc | Cd69 | Rps12 | Jup | Cybb | Epcam | Gimap9 | Hist1h2bc |
| Rpl11 | Rps18 | Mbnl3 | Tek | Cst3 | Hspa1b | Rps4x | Perp | Phf11b | Parm1 | Ccnd2 | 1810058I24Rik |
| Rpl32 | Rpl37 | Ceacam3 | Nova2 | Dcn | Rel | Rps23 | Fn1 | Dock10 | Cep170b | Ptpn22 | Rsu1 |
| Rpl19 | Cdr2 | Sema3f | Kif26a | Selenop | Lamb3 | Rpsa | Fthl17a | Ltb4r1 | Ptprk | Ets1 | Sh3bgrl3 |
| Rps10 | Ppia | Flt1 | Hspa12b | Aprt | Stk17b | Rpl34 | Nrk | BC028528 | Srd5a1 | Grap2 | Hacd4 |
| Rps14 | Rpl8 | Trp53i11 | Icam2 | Klf4 | Cd52 | Fth1 | Kazn | Epsti1 | Krt8 | Ppp1r16b | Tsc22d1 |

| **Invasive**  **Sphongio-trophoblast2** | **Endodermal**  **Cell** | **Unknown** | **Sphongio-trophoblast** | **Decidual**  **Trophoblast** | **Decidual1** | **Invasive**  **Sphongio-trophoblast1** | **Stromal** | **Macrophage** | **Monocyte** | **Granulocyte** | **Decidual2** |
| --- | --- | --- | --- | --- | --- | --- | --- | --- | --- | --- | --- |
| F11 | Kng1 | Bub1 | Dlx3 | Cuzd1 | Dcn | Fbln7 | Col1a1 | C1qc | Ms4a6c | S100a9 | Pgr |
| Slit1 | Spink1 | Pclaf | Gjb2 | Serpina1e | Gpx3 | Ctsr | Col1a2 | C1qa | Cybb | Retnlg | Slco5a1 |
| Hand1 | Apoa4 | Rad51 | B230312C02Rik | Guca2b | Ly6c1 | Psg28 | Itm2a | C1qb | Clec4a3 | Hdc | Hoxa10 |
| Doxl2 | Fgb | Hist1h1b | Sgk2 | Akr1c18 | Ctla2a | Prl7a2 | Col6a3 | Fcrls | Ms4a6d | Hcar2 | Tnfrsf11b |
| Sfmbt2 | Serpina1a | Ckap2l | Snap91 | Sult1d1 | Ctsk | Psg26 | Col6a1 | Ms4a7 | Fcgr1 | Csf3r | Masp1 |
| Psg17 | Bex4 | Ccna2 | AU018091 | Wfdc2 | Gatm | Ctsm | Col6a2 | Aif1 | F10 | Trem1 | Hoxa9 |
| Ctsr | Apom | Cenpf | Lrp8 | Aqp8 | Srgn | Psg18 | Postn | Mrc1 | Ctss | Mmp9 | Matn2 |
| Psg25 | Fga | Cdca3 | Lipg | Tmprss4 | Ftl1 | Psg25 | Col5a1 | C3ar1 | Pld4 | S100a8 | Mrgprg |
| Rab15 | Spp2 | Cdca8 | Tfrc | Sftpd | Lbp | Prl2b1 | Fbn1 | Apoe | Mpeg1 | Slc7a11 | Slc25a29 |
| Grhl2 | Bex2 | Birc5 | Slco4a1 | Fxyd3 | Rbp4 | Taf7l | Lox | Ms4a6d | Ifi207 | Il1b | Hoxa11 |
| Tfap2c | Fgg | Prc1 | Caskin1 | Abcb1b | Cyp11a1 | Psg19 | Ptn | Ccl12 | Itgb2 | Wfdc21 | Atp8b1 |
| Fetub | Smlr1 | Top2a | Sept4 | S100g | Lgals3 | Psg27 | Col5a2 | Clec4a1 | Cd300c2 | Ccr1 | 4930486L24Rik |
| Gjb3 | Serpina1b | Asf1b | Amot | Kap | Mustn1 | Psg21 | Pcolce | Stab1 | Slc11a1 | Lmnb1 | Cysltr2 |
| Psg22 | Ttr | Cdk1 | Zim1 | Cldn10 | Mif | Ceacam13 | Ccdc80 | Ctss | Fyb | Clec4d | Sorbs2 |
| Ceacam3 | Psca | Cdkn3 | Rassf6 | Spint2 | Dbi | Tpbpb | Cdh11 | Adgre1 | Chil3 | G0s2 | Cd109 |
| Psg19 | F2 | Tpx2 | Zdbf2 | Ltf | Cd63 | Ceacam14 | Mfap2 | Ms4a6c | Igsf6 | Clec4e | Eva1c |
| Prodh | Serpina1c | Aurkb | Peg3 | Prap1 | Htra3 | Tpbpa | Cd248 | Aoah | Lair1 | Cxcl2 | Sez6l |
| Eva1a | Cfi | Ccnb1 | Car4 | C3 | Chchd10 | Creg1 | Islr | Tyrobp | Lyz2 | Il1r2 | Cldn11 |
| Pou3f1 | 4933402E13Rik | Ndc80 | Fxyd6 | F3 | S100a1 | Psg17 | Loxl1 | Fcgr1 | Ms4a4c | Acod1 | Mmp11 |
| Plekhh1 | Apoc1 | Ccnb2 | Kcnq1ot1 | Epcam | S100a11 | Prl8a8 | Bgn | Ms4a6b | Mafb | Lrg1 | Cxcl14 |
| Phldb3 | Alb | Hmmr | Macf1 | Krt18 | Psmd8 | Ceacam11 | Ltbp4 | Ctsc | Lst1 | Samsn1 | Adm |
| Slc4a8 | Fbp2 | Spc24 | Gm47283 | Napsa | Tceal9 | Ceacam5 | Adamts2 | Fcgr3 | Spi1 | Hp | Cped1 |
| Pramef12 | Apoa1 | Sgo2a | Bbx | Krt7 | Txn1 | Ceacam12 | Rbms3 | Gas6 | Ly6c2 | Tyrobp | Pla2g2e |
| Adamts15 | Afp | Dlgap5 | Arhgef6 | Srd5a1 | Dmkn | Prl8a9 | Col3a1 | Fcer1g | Ms4a6b | Slpi | Scnn1b |
| Slc22a5 | Amn | Tacc3 | Ppp2r3a | Tgfb2 | Ybx1 | 1600012P17Rik | Crispld2 | P2ry6 | Tyrobp | AC110211.1 | Inhba |
| Amot | Kng2 | Mki67 | Peg10 | Dhcr24 | Fabp4 | Cts3 | Lum | Pf4 | Gngt2 | Cxcr2 | Cyp11a1 |
| Egfr | Rbp2 | Racgap1 | Eps8l2 | Hsd11b1 | Sptssa | Slc38a4 | Adam12 | Npl | Laptm5 | Cd14 | Erv3 |
| Cpxm1 | Entpd2 | Anln | Dlg5 | Ctsb | H2-Q7 | Prl3b1 | Mmp2 | Ifi207 | Fcer1g | Mxd1 | Pamr1 |
| Zim1 | Rbp4 | Incenp | Neat1 | Tspan8 | Cyb5a | Pappa2 | Loxl2 | Ccl9 | Hck | Mcemp1 | Sfrp5 |
| Prl8a1 | Apoa2 | Smc2 | Usp48 | Sgms2 | Serpinb6a | Psg23 | Fstl1 | Mafb | Alox5ap | H2-Q10 | Wt1 |
| Srgap1 | Bst1 | Kif23 | Helz | Csf1 | Serf2 | Prl8a6 | Cd34 | Lyz2 | Irf5 | Nlrp3 | Btbd3 |
| Htr2b | Gipc2 | Cks1b | Ahnak | Cd24a | S100a10 | Psg16 | Fzd2 | Lgmn | Cyp4f18 | Stfa2l1 | Aif1l |
| Il2rb | Folr1 | Cit | Plagl1 | Krt8 | Ramp1 | Prl3a1 | Spon1 | F13a1 | Ly86 | Il1f9 | A2m |
| Arhgef16 | Cldn7 | Cenpw | Plekhh1 | Cldn7 | S100a6 | Prl8a1 | Pde3a | Fcgr2b | Cd300a | Lilr4b | Antxr1 |
| Ggt1 | Cyp11a1 | Hist1h2ae | Bclaf3 | Mal | B2m | Flt1 | Fbln2 | Slco2b1 | Cd53 | Cd300lf | Acot2 |
| Nppc | Podxl | Slamf9 | Mark3 | Ctsl | Sbsn | Fthl17a | Mgp | Fyb | Cxcr4 | Asprv1 | Prrx1 |
| Slc38a4 | Fabp3 | Stmn1 | Luc7l | Gm42418 | H2-K1 | Cstb | Prelp | Dab2 | Slc15a3 | Ifitm1 | Slc6a12 |
| Mdfi | 1300017J02Rik | Tbxas1 | Tns1 | Gpx3 | Prdx1 | Krt8 | Adgra2 | Tnfaip8l2 | Ncf2 | Plek | Nrn1l |
| Prl3a1 | Proc | Dck | Ppp1r9a | Gsto1 | Hist1h2bc | Tmsb10 | Timp1 | Igf1 | Tlr2 | Ankrd33b | Aoc1 |
| Efna1 | Cldn3 | AU020206 | Hnrnpr | Cryab | Serping1 | Pdia6 | Grb10 | Ly86 | Ptprc | Slfn4 | Dtna |
| Psg29 | Cryl1 | Clec5a | Ubr4 | Gng12 | Cpe | Ceacam3 | Lhfp | Clec4a2 | Coro1a | Gm5483 | Vldlr |
| Ceacam5 | Rhox5 | Cenpa | Kitl | Sec11a | H2-D1 | Krt18 | Tgfb1i1 | Cyth4 | Klra2 | Il1rn | Lpar1 |
| Derl3 | Plet1 | Clec4a2 | Acvr2b | Ly6e | Cryab | Gm42418 | Lpar1 | Selenop | Cd52 | Arg2 | Cpe |
| Igsf9 | Clu | Kcnn4 | Plekha7 | Tmed3 | Gpx4 | Psg29 | Mmp23 | Grn | Itgal | Cd300ld | Htra3 |
| Efs | Cldn4 | C1qb | Mycbp2 | Rplp0 | Des | Calr | Bicc1 | Ifi27l2a | Cytip | C5ar1 | Ctsk |
| Tmem108 | Peg10 | Ly86 | Ankrd17 | Ssr4 | Npc2 | Maged1 | Col18a1 | Evi2a | Lyn | Pglyrp1 | Hhipl1 |
| Yipf2 | Trf | Smc4 | Fam90a1b | Bst2 | Prl8a2 | Rhox9 | Dcn | Nrros | Plbd1 | Adam8 | Atp6v0d2 |
| Xylt1 | Fcgrt | C1qc | Tns4 | Tmbim6 | H2-T22 | Cited2 | Scarf2 | Pld4 | Rel | Rnf149 | Aebp1 |
| C1qtnf1 | Gpc3 | Dctpp1 | Slc38a1 | Mkrn1 | Gnas | Hspa5 | Sdc2 | Tbxas1 | Cx3cr1 | Cebpb | Mob3b |
| Dtx1 | C2 | Topbp1 | Zc3h7a | Srgn | Myl6 | Sct | Gja1 | Rassf4 | Csf1r | Alox5ap | Mustn1 |

**Supplementary Table 3:** List of Differentially Expressed Genes between CON and AP Samples in Bulk-RNAseq.

| **Gene** | **log2 Fold Change** | **P value** | **padj** | **Up/Dn** |
| --- | --- | --- | --- | --- |
| Ceacam9 | 3.502115004 | 4.40E-46 | 7.13E-42 | 1 |
| Spire2 | -2.636867173 | 2.79E-33 | 1.51E-29 | -1 |
| Endou | -2.462868789 | 1.80E-28 | 7.29E-25 | -1 |
| Ly6g6c | -2.467854104 | 7.01E-26 | 1.62E-22 | -1 |
| Igfbp6 | -2.419988983 | 3.65E-22 | 4.55E-19 | -1 |
| Cpne5 | 2.677880012 | 4.23E-19 | 2.74E-16 | 1 |
| Spag5 | 2.000121105 | 9.10E-18 | 4.91E-15 | 1 |
| Hbb-y | 5.264286384 | 6.74E-13 | 1.19E-10 | 1 |
| Chrdl1 | 2.251091741 | 4.70E-12 | 6.56E-10 | 1 |
| Podn | -2.074471353 | 2.88E-11 | 3.05E-09 | -1 |
| Cyp1a1 | 4.26312552 | 5.91E-11 | 5.77E-09 | 1 |
| Mlc1 | 2.2743729 | 7.19E-11 | 6.73E-09 | 1 |
| Adamtsl2 | -2.136541315 | 9.98E-11 | 8.93E-09 | -1 |
| Il1f6 | 2.137169114 | 5.28E-10 | 3.89E-08 | 1 |
| A730036I17Rik | -2.395163595 | 9.49E-10 | 6.43E-08 | -1 |
| Nyx | -2.044845166 | 1.40E-09 | 9.06E-08 | -1 |
| Hba-x | 3.917783038 | 1.62E-08 | 7.47E-07 | 1 |
| 4933424G05Rik | -2.951059368 | 1.84E-08 | 8.43E-07 | -1 |
| Tmem145 | -2.833661854 | 2.35E-08 | 1.04E-06 | -1 |
| Kcnt1 | -3.054698206 | 2.53E-08 | 1.10E-06 | -1 |
| Fcgbp | -2.332430278 | 3.02E-08 | 1.27E-06 | -1 |
| Slc1a2 | 2.385717579 | 4.26E-08 | 1.69E-06 | 1 |
| Itk | 2.035736652 | 6.08E-08 | 2.30E-06 | 1 |
| Hamp | 2.912247765 | 7.64E-08 | 2.78E-06 | 1 |
| Nppb | -2.46220998 | 2.62E-07 | 7.75E-06 | -1 |
| Pax2 | 2.200188993 | 5.03E-07 | 1.37E-05 | 1 |
| Edaradd | 2.378578447 | 5.43E-07 | 1.45E-05 | 1 |
| Slc13a5 | -2.674433974 | 6.74E-07 | 1.73E-05 | -1 |
| Capn9 | -5.783929429 | 1.36E-06 | 3.07E-05 | -1 |
| Wisp2 | -2.028264515 | 1.76E-06 | 3.78E-05 | -1 |
| Btc | -2.841656157 | 1.99E-06 | 4.20E-05 | -1 |
| Hbb-bh1 | 3.261312391 | 2.47E-06 | 4.94E-05 | 1 |
| Eln | -2.145482815 | 3.36E-06 | 6.39E-05 | -1 |
| Ccl3 | 3.179805047 | 5.56E-06 | 9.66E-05 | 1 |
| Bhlhe22 | -2.33087279 | 6.14E-06 | 0.000104 | -1 |
| Cdh26 | -2.25931689 | 8.24E-06 | 0.000134 | -1 |
| Cabp7 | 3.306379228 | 9.39E-06 | 0.00015 | 1 |
| Gm4956 | -2.021361945 | 1.40E-05 | 0.000207 | -1 |
| Saa1 | -2.703222988 | 1.49E-05 | 0.000218 | -1 |
| Ptger2 | -2.928882323 | 1.61E-05 | 0.000231 | -1 |
| Allc | 3.189174426 | 1.71E-05 | 0.000244 | 1 |
| Nrg1 | -2.463819387 | 1.82E-05 | 0.000258 | -1 |
| Chrm2 | -2.635305991 | 2.37E-05 | 0.000324 | -1 |
| Olfr224 | 2.451258005 | 2.71E-05 | 0.000362 | 1 |
| Ace3 | 2.610194927 | 2.82E-05 | 0.000376 | 1 |
| C030034L19Rik | -2.403230853 | 3.08E-05 | 0.000403 | -1 |
| Cd5l | 3.707803245 | 3.27E-05 | 0.000422 | 1 |
| Adamts16 | -3.027309329 | 4.61E-05 | 0.00056 | -1 |
| Mchr1 | -2.011388261 | 4.74E-05 | 0.00057 | -1 |
| Slc7a15 | -2.534676425 | 4.77E-05 | 0.000572 | -1 |
| Marco | 3.74822801 | 5.40E-05 | 0.000632 | 1 |
| Jph4 | -2.306532234 | 5.49E-05 | 0.000642 | -1 |
| Tmem72 | 2.953227424 | 5.85E-05 | 0.00068 | 1 |
| Irg1 | 3.080289749 | 8.76E-05 | 0.000952 | 1 |
| Cdh17 | -2.162348896 | 8.88E-05 | 0.000962 | -1 |
| Ssu2 | 2.052312568 | 0.000156 | 0.001525 | 1 |
| Phyhip | -2.346048836 | 0.000158 | 0.001539 | -1 |
| Ngfr | 2.935827175 | 0.000178 | 0.001689 | 1 |
| E330021D16Rik | -3.403585726 | 0.000185 | 0.00174 | -1 |
| Nol4 | 2.145417871 | 0.000197 | 0.001821 | 1 |
| Abca13 | -2.57676083 | 0.000226 | 0.002038 | -1 |
| Stoml3 | 2.222816227 | 0.000366 | 0.003019 | 1 |
| Rxfp1 | -2.291913854 | 0.000398 | 0.003223 | -1 |
| Cd300lf | 2.012983892 | 0.000406 | 0.003272 | 1 |
| Slc9a3 | -3.215330783 | 0.000406 | 0.003274 | -1 |
| 1700013H16Rik | -3.49196229 | 0.000424 | 0.003383 | -1 |
| Tmem27 | -2.461735889 | 0.000447 | 0.003534 | -1 |
| Erich4 | -2.299249897 | 0.000474 | 0.003721 | -1 |
| Tph1 | -3.570235337 | 0.000491 | 0.003824 | -1 |
| Pnoc | -2.415628698 | 0.000674 | 0.004918 | -1 |
| Bcl2a1b | 2.084352577 | 0.000721 | 0.005189 | 1 |
| Ntrk1 | -2.229902745 | 0.000727 | 0.005215 | -1 |
| Cpa5 | 2.272845067 | 0.000829 | 0.005782 | 1 |
| Shisa6 | -2.568333432 | 0.000914 | 0.006243 | -1 |
| Dupd1 | -2.09277477 | 0.001062 | 0.007026 | -1 |
| Rsph4a | -2.01121641 | 0.001131 | 0.007399 | -1 |
| Dgkb | -2.076814223 | 0.0012 | 0.00774 | -1 |
| Cxcl2 | 3.10204939 | 0.001218 | 0.007828 | 1 |
| Tac2 | 2.423325517 | 0.001388 | 0.008666 | 1 |
| Aplnr | 2.037908991 | 0.001724 | 0.010279 | 1 |
| Lrat | -2.917012431 | 0.00206 | 0.011829 | -1 |
| Scg5 | -2.378773671 | 0.002088 | 0.011944 | -1 |
| Hao2 | -2.952814412 | 0.002228 | 0.012595 | -1 |
| Prodh2 | -2.392796594 | 0.002537 | 0.013925 | -1 |
| BC021785 | -2.344171307 | 0.002657 | 0.014452 | -1 |
| Rimbp2 | -2.436076756 | 0.002678 | 0.014545 | -1 |
| Urah | -2.007041172 | 0.002927 | 0.015565 | -1 |
| Spic | 2.880602761 | 0.002992 | 0.015827 | 1 |
| Cidec | -2.215408754 | 0.003058 | 0.016082 | -1 |
| Pgm5 | -2.120460757 | 0.003403 | 0.017486 | -1 |
| Fancd2os | 2.025411851 | 0.003413 | 0.017523 | 1 |
| Vmn2r121 | -2.724728768 | 0.003586 | 0.01821 | -1 |
| Accsl | 2.147162205 | 0.003863 | 0.019326 | 1 |
| Khdc3 | -2.773572205 | 0.003967 | 0.019765 | -1 |
| Dmrt3 | -2.057619471 | 0.004189 | 0.020558 | -1 |
| Sec14l3 | -2.683638339 | 0.004485 | 0.021721 | -1 |
| Ubtfl1 | -3.339385005 | 0.004541 | 0.021919 | -1 |
| Lrrc74b | -2.241494593 | 0.005441 | 0.025252 | -1 |
| Mpped1 | 2.345948974 | 0.005444 | 0.025262 | 1 |
| Camp | 2.112845112 | 0.005452 | 0.025291 | 1 |
| Pou6f2 | 2.22001349 | 0.005527 | 0.02553 | 1 |
| Ccl4 | 2.30755366 | 0.005544 | 0.025592 | 1 |
| Gm20756 | -2.259447881 | 0.005724 | 0.026241 | -1 |
| Megf10 | 2.552202427 | 0.006033 | 0.027264 | 1 |
| Gm21269 | -4.113746067 | 0.006203 | 0.027897 | -1 |
| Sectm1b | -2.105375091 | 0.006833 | 0.030087 | -1 |
| Asb11 | -2.209042968 | 0.007026 | 0.030743 | -1 |
| Rhox13 | 2.993075521 | 0.007086 | 0.030937 | 1 |
| Th | 2.362361138 | 0.007574 | 0.032518 | 1 |
| Adgra1 | -2.46353346 | 0.007615 | 0.032649 | -1 |
| Dppa1 | -3.382114778 | 0.007768 | 0.033148 | -1 |
| Mcoln3 | -2.109154364 | 0.007968 | 0.033777 | -1 |
| Gpr84 | 3.070962452 | 0.008752 | 0.036181 | 1 |
| Xist | 2.668786018 | 0.008779 | 0.036267 | 1 |
| Myt1l | 2.360164963 | 0.00881 | 0.036358 | 1 |
| Sapcd2 | -2.197029065 | 0.008917 | 0.036711 | -1 |
| Mt4 | 2.63503316 | 0.009653 | 0.038943 | 1 |
| Vdr | -2.06996377 | 0.010523 | 0.041503 | -1 |

**Supplementary Table 4:** Antibodies used in flow cytometry analysis.

| **Cell Marker** | **Fluorochrome** | **Clone** | **Company** | **Cat No** |
| --- | --- | --- | --- | --- |
| Live/Dead | DAPI |  | Life Technologies | L23105 |
| CD45 | Super Bright 436 (SB436) | 30-F11 | e Bioscience | 62-0451-82 |
| Ly-6G/Ly-6C | Super Bright 600 (SB600) | RB6-8C5 | e Bioscience | 63-5931-82 |
| B220 (CD45R) | Super Bright 645 (SB645) | RA3-6B2 | e Bioscience | 64-0452-82 |
| NK1.1 | Super Bright 702 (SB702) | PK136 | e Bioscience | 67-5941-82 |
| CD11c | Super Bright 780 (SB780) | N418 | e Bioscience | 78-0114-82 |
| CD11b | Alexa Fluor 488 | M1/70 | e Bioscience | 53-0112-82 |
| CD3 | PerCP-eFluor 710 | 17A2 | e Bioscience | 46-0032-82 |
| F4/80 | PE | BM8 | e Bioscience | 12-4801-82 |
| CD8 | PE-Cyanine7 | 53-6.7 | e Bioscience | 25-0081-82 |
| CD206 | APC | MR6F3 | e Bioscience | 17-2061-82 |
| CD4 | APC-eFluor 780 |  | e Bioscience | 47-0041-82 |
